# Decitabine alters the expression of *Mecp2* isoforms *via* dynamic DNA methylation at the *Mecp2* regulatory elements in neural stem cells

**DOI:** 10.1186/2040-2392-4-46

**Published:** 2013-11-15

**Authors:** Vichithra R B Liyanage, Robby M Zachariah, Mojgan Rastegar

**Affiliations:** 1 Regenerative Medicine Program, Department of Biochemistry and Medical Genetics, Faculty of Medicine, University of Manitoba, Rm. 627, Basic Medical Sciences Bldg., 745 Bannatyne Avenue, Winnipeg, Manitoba R3E 0J9, Canada

**Keywords:** Epigenetics, *Mecp2e1*, *Mecp2e2*, Decitabine/5-aza-2′-deoxycytidine, DNA methylation, Autism

## Abstract

**Background:**

Aberrant MeCP2 expression in brain is associated with neurodevelopmental disorders including autism. In the brain of stressed mouse and autistic human patients, reduced MeCP2 expression is correlated with *Mecp2*/*MECP2* promoter hypermethylation. Altered expression of MeCP2 isoforms (MeCP2E1 and MeCP2E2) is associated with neurological disorders, highlighting the importance of proper regulation of both isoforms. While known regulatory elements (REs) within the *MECP2/Mecp2* promoter and intron 1 are involved in *MECP2/Mecp2* regulation, *Mecp2* isoform-specific regulatory mechanisms are unknown*.* We hypothesized that DNA methylation at these REs may impact the expression of *Mecp2* isoforms.

**Methods:**

We used a previously characterized *in vitro* differentiating neural stem cell (NSC) system to investigate the interplay between *Mecp2* isoform-specific expression and DNA methylation at the *Mecp2* REs. We studied altered expression of *Mecp2* isoforms, affected by global DNA demethylation and remethylation, induced by exposure and withdrawal of decitabine (5-Aza-2′-deoxycytidine). Further, we performed correlation analysis between DNA methylation at the *Mecp2* REs and the expression of *Mecp2* isoforms after decitabine exposure and withdrawal.

**Results:**

At different stages of NSC differentiation, *Mecp2* isoforms showed reciprocal expression patterns associated with minor, but significant changes in DNA methylation at the *Mecp2* REs. Decitabine treatment induced *Mecp2e1*/MeCP2E1 (but not *Mecp2e2*) expression at day (D) 2, associated with DNA demethylation at the *Mecp2* REs. In contrast, decitabine withdrawal downregulated both *Mecp2* isoforms to different extents at D8, without affecting DNA methylation at the *Mecp2* REs. NSC cell fate commitment was minimally affected by decitabine under tested conditions. Expression of both isoforms negatively correlated with methylation at specific regions of the *Mecp2* promoter, both at D2 and D8. The correlation between intron 1 methylation and *Mecp2e1* (but not *Mecp2e2*) varied depending on the stage of NSC differentiation (D2: negative; D8: positive).

**Conclusions:**

Our results show the correlation between the expression of *Mecp2* isoforms and DNA methylation in differentiating NSC, providing insights on the potential role of DNA methylation at the *Mecp2* REs in *Mecp2* isoform-specific expression. The ability of decitabine to induce *Mecp2e1*/MeCP2E1, but not *Mecp2e2* suggests differential sensitivity of *Mecp2* isoforms to decitabine and is important for future drug therapies for autism.

## Background

**Figure 1:**
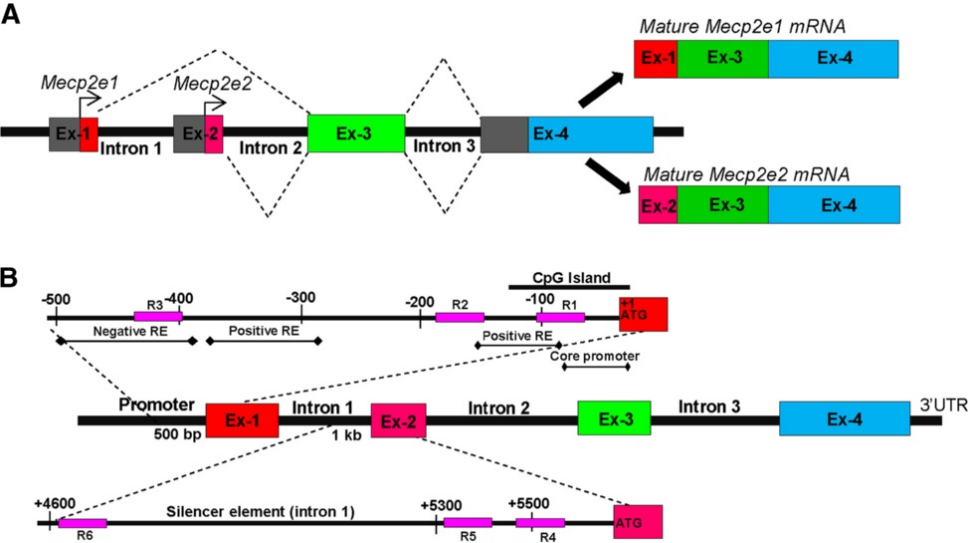
**Schematics of the Methyl CpG binding protein 2 gene (*Mecp2*), *Mecp2e1*/*e2 *transcripts, and known regulatory elements (REs). (A)** Generation of MeCP2 isoforms by alternative splicing; mature *Mecp2e1* transcripts comprise of exons 1, 3, and 4. Mature *Mecp2e2* transcripts comprise of exons 2, 3, and 4 (adapted from [4,11]). Exons are denoted as Ex. **(B)** Regulatory elements of the *MECP2/Mecp2* gene. The *MECP2/Mecp2* gene is reported to be regulated by negative and positive REs within the promoter and a silencer element within the intron 1 (information extracted from [12,13]). For our studies we selected a 500-bp region in the promoter upstream of the exon 1 and a 1-kb region in the intron 1 upstream of the exon 2. Each sequence was divided into three regions, R1 to R3 in the *Mecp2* promoter and R4 to R6 in the intron 1. Note that there are no CpG dinucleotides in the mouse genomic sequence between R5 and R6.

Methyl CpG Binding Protein 2 (MeCP2) is a key transcriptional regulator in the brain [1]. *MECP2* mutations and expression deficits result in a broad range of neurodevelopmental disorders, including Rett syndrome (RTT) and autism spectrum disorders [2,3]. In mice (*Mecp2*) and humans (*MECP2*), alternative splicing of a single gene leads to the generation of two protein isoforms MeCP2E1 and MeCP2E2 (mature transcripts for *Mecp2e1* and *Mecp2e2* are shown in Figure 1A) [4,5]. We and others have shown differential expression of the two *Mecp2*/MeCP2 isoforms in mouse brain [5-7]. Recent studies suggest that MeCP2E1 is the most relevant isoform for RTT pathology [8,9]. Moreover, overexpression of *Mecp2e2*, but not *Mecp2e1*, promotes neuronal cell death [10], implicating the importance of proper regulation of both *Mecp2* isoforms in the brain.

In RTT mouse models, transgenic expression of either *Mecp2* isoform can rescue RTT phenotypes to different extents [14,15]. However, gene therapy delivery of *MECP2* into the affected cells or drug therapies to induce *MECP2* expression has to be carried out with caution, as even mild overexpression of MeCP2 can lead to progressive neurological disorders [16,17]. Currently, limited knowledge exists on *MECP2/Mecp2* regulation*,* with no specific knowledge on possible differential *MECP2/Mecp2* isoform-specific regulatory mechanisms.

*MECP2/Mecp2* gene expression is known to be regulated by regulatory elements (REs) within the promoter and a silencer element within the *Mecp2* intron 1 [12,13,18] (Figure 1B). Implying the role of DNA methylation in *MECP2* regulation, reduced *MECP2* expression in the brains of male autistic patients correlates with human *MECP2* promoter hypermethylation [2,19]. Moreover, reduced *Mecp*2 expression in the postnatal murine brain in response to early maternal separation and stress is associated with hypermethylation of the mouse *Mecp2* promoter [20]. However, possible differential impact of DNA methylation on *MECP2*/*Mecp2* isoforms is currently unknown. DNA methylation is a major epigenetic modification that controls gene expression without affecting the underlying DNA sequences (reviewed in [21,22]). DNA methylation at the cytosine residues (5-methylcytosine (5mC)) of the CpG dinucleotides is carried out by DNA methyltransferases (DNMT) and is generally considered to be a repressive epigenetic modification [1,23]. Conversely, 5-hydroxymethylcytosine (5hmC), which is generated by oxidation of 5mC by TET proteins is generally considered to be an active epigenetic mark [24,25]. Promoter methylation is mostly associated with gene silencing [26], while DNA methylation at both intronic and exonic regions are shown to correlate with isoform-specific transcription by alternative splicing or by utilizing alternate promoters [27,28].

Treatment with DNA demethylating agents or *DNMT*/*Dnmt* inhibitors such as decitabine (also called 5-Aza-2′-deoxycytidine) is a commonly used method to study the role of DNA methylation in gene expression [29,30]. While exposure to decitabine results in DNA demethylation, its subsequent withdrawal causes remethylation or methylation reprogramming [29], providing an excellent platform to uncover the role of DNA methylation in gene expression.

*In vitro* differentiation of neural precursor cells/neural stem cells (NSC) into different brain cell types is utilized as an acceptable model system to mimic the *in vivo* neural development [31-36]. Previously, we used a similar *in vitro* NSC differentiation system to report the first preclinical *MECP2* isoform-specific gene therapy vectors, for future gene therapy applications in Rett syndrome [35]. Further, we introduced differentiating NSC as a suitable *in vitro* model to study the expression and function of developmentally important genes such as *Meis1* in neural development [37]. In the current study, we used this previously characterized system to study the expression and regulation of *Mecp2* isoforms during NSC differentiation.

Investigation of *MECP2*/MeCP2 expression and function in neurodevelopmental disorders has been the focus of intensive research. However, despite the critical importance of precisely controlled levels of MeCP2 expression in the brain, the underlying regulatory mechanisms have been understudied. Here, we report the correlation between the expression of *Mecp2* isoforms and DNA methylation patterns at the *Mecp2* REs at different stages of NSC differentiation. Further, we demonstrate the effect of dynamic changes in DNA methylation induced by exposure and withdrawal of decitabine on the expression of *Mecp2/*MeCP2 isoforms.

## Methods

### Ethics statement

All experiments were performed in accordance with the standards of the Canadian Council on Animal Care with the approval of the Office of Research Ethics of University of Manitoba. All experimental procedures were reviewed and approved (protocol number 12–031) by the University of Manitoba Bannatyne Campus Protocol Management and Review Committee.

### Neural stem cell isolation, culture and differentiation

Embryonic mouse forebrain-derived NSC were isolated from the forebrains of CD-1 mice at embryonic day (E) 14.5 and were cultured according to previously described methods [35,37]. Briefly, dissected forebrain tissues were mechanically homogenized in NSC media DMEM/F12 1:1 (Wisent, Quebec, Canada) containing HEPES, glutamine, antibiotic/antimycotic, glucose, recombinant human epidermal growth factor (rhEGF) (Sigma Aldrich, Oakville, Ontario, Canada, 20 ng/ml), basic fibroblast growth factor (bFGF) (Upstate (Millipore), Billerica, MA, USA, 20 ng/ml), heparin (Sigma Aldrich, Oakville, Ontario, Canada, 2 μg/ml) and hormone mix. Dissociated single cells were plated at a density of 10^5^ cells/cm^2^ in NSC media. The media were refreshed every 48 h and cells were cultured under these conditions for 7 days to generate neurospheres. Primary neurospheres were gently dissociated to single cells by accutase treatment. Dissociated cells were plated on plates coated with growth factor-reduced matrigel (BD Biosciences, Mississauga, Ontario, Canada) at a density of 10^5^ cells/cm^2^ in DMEM (GIBCO, Life Technologies Inc, Burlington, Ontario, Canada) and 10% Fetal Bovine Serum (Invitrogen, Life Technologies Inc, Burlington, Ontario, Canada) in the absence of rhEGF and bFGF. Cells were differentiated for 8 days, reported to be sufficient for differentiation of neuronal and glial cells [35,37], and media were changed every other day.

### Decitabine treatment

At the onset of differentiation on day zero (D0), dissociated NSC were treated with 2.5 μM decitabine for 48 h. After two days (D2), the media were replaced with fresh media that was refreshed every other day for an extra 6 days (until D8). Control cells were cultured under similar experimental conditions, in the absence of decitabine.

### Quantitative measurement of male/female contribution

Genomic DNA from neural stem cells at D0, D2, D8 and decitabine-treated cells were extracted using the DNeasy Blood and Tissue kit (Qiagen, Ontario, Toronto, Canada), as per manufacturer’s instructions. The contribution of male and female sexes were determined by semiquantitative PCR-based amplification of *Sry* (sex-determining region protein gene in the Y chromosome) and *Il3* (autosomal gene as an internal control) genes, as described previously [38], using the primers listed in Table 1. The PCR program consisted of an initial denaturation at 95°C for 4.5 minutes, followed by 33 cycles of 95°C for 35 s, 50°C for 1 minute, 72°C for 1 minute, and a final extension step at 72°C for 5 minutes. The amplified products were run on 1.5% agarose gel and the bands were visualized by ethidium bromide staining. The *Sry* and *Il3* PCR products were identified based on the corresponding sizes (*Sry:* 402 bp, and *Il3*: 544 bp). Intensity of the corresponding bands was quantified using Adobe Photoshop CS5 software. The contribution of either sex was further determined by quantitative reverse transcription PCR (qRT-PCR) for *Xist* (X-inactive specific transcript) gene, as previously described, with minor modifications [39]. The PCR program for *Xist* included an initial denaturation at 95°C for 5 minutes; followed by 35 cycles of 95°C for 30 s, 53°C for 30 s, 72°C for 30 s, and 78°C for 30 s.

**Table 1:** List of primers used for PCR

**Gene**	**Direction**	**Sequence**	**Reference**
***Sry***	Forward	5′-TGGGACTGGTGACAATTGTC-3′	[38]
	Reverse	5′-GAGTACAGGTGTGCAGCTCT-3′	
***Il3***	Forward	5′-GGGACTCCAAGCTTCAATCA-3′	
	Reverse	5′-GGAGGAGGAAGAAAAGCAA-3′	

### Quantitative reverse transcription polymerase chain reaction

RNeasy Mini Kit (Qiagen, Ontario, Toronto, Canada) was used for RNA extraction as per the manufacturer’s protocol. Preparation of cDNA and qRT-PCR were carried out as described previously [40-42]. Transcript levels of *Mecp2* (total), *Mecp2e1* [NCBI: NM_001081979.1], *Mecp2e2* [NCBI: NM_010788.3], *Dnmt* genes (*Dnmt1, Dnmt3a* and *Dnmt3b*), neuronal genes (*Tubulin III* (*Tub III*), *NeuN*), astrocytic genes (*Gfap*, *S100b*), and oligodendrocyte-specific genes (*Cnpase*, *Mbp*) were examined by using gene-specific primers (Table 2), as described previously [37,43]. The relative expression and fold changes were calculated as described previously [37]. Two-way analysis of variance (ANOVA) and the Student *t*-test were used to calculate significant differences between untreated control and decitabine-treated samples.

**Table 2:** List of primers used for qRT-PCR

**Gene**	**Direction**	**Sequence**	**Reference**
***Mecp2 *** **(total)**	Forward	5′-GGTAAAACCCGTCCGGAAAATG-3′	[4]
	Reverse	5′-TTCAGTGGCTTGTCTCTGAG-3′	
***Mecp2e1***	Forward	5′-AGGAGAGACTGGAGGAAAAGT-3′	[5]
	Reverse	5′-CTTAAACTTCAGTGGCTTGTCTCTG-3′	
***Mecp2e2***	Forward	5′-CTCACCAGTTCCTGCTTTGATGT-3′	
	Reverse	5′-CTTAAACTTCAGTGGCTTGTCTCTG-3′	
***Tubulin III (Tub III*)**	Forward	5′-TCAGCGATGAGCACGGCATA-3′	[37]
	Reverse	5′-CACTCTTTCCGCACGACATC-3′	
***Gfap***	Forward	5′-GCTCACAATACAAGTTGTCC-3′	
	Reverse	5′-ACCTAATTACACAGAGCCAGG-3′	
***Gapdh***	Forward	5′-AACGACCCCTTCATTGAC-3′	[43]
	Reverse	5′-TCCACGACATACTCAGCAC-3′	
***NeuN***	Forward	5′-GGCAATGGTGGGACTCAAAA-3′	[44]
	Reverse	5′-GGGACCCGCTCCTTCAAC-3′	
***S100b***	Forward	5′-GCTGACCACCATGCCCCTGTAG-3′	[45]
	Reverse	5′-CTGGCCATTCCCTCCTCTGTC-3′	
***Mbp***	Forward	5′-GGCACGCTTTCCAAAATCT-3′	[46]
	Reverse	5′-CCATGGGAGATCCAGAGC-3′	
***Cnpase***	Forward	5′-CATCCTCAGGAGCAAAGGAG-3′	[47]
	Reverse	5′-TGAATAGCGTCTTGCACTCG-3′	
***Dnmt1***	Forward	5′-AGGGAAAAGGGAAGGGCAAG-3′	[48]
	Reverse	5′-AGAAAACACATCCAGGGTCCG-3′	
***Dnmt3a***	Forward	5′-CAGCGTCACACAGAAGCATATCC-3′	
	Reverse	5′-GGTCCTCACTTTGCTGAACTTGG-3′	
***Dnmt3b***	Forward	5′-CCTGCTGAATTACTCACGCCCC-3′	
	Reverse	5′-GTCTGTGTAGTGCACAGGAAAA-3′	
***Xist***	Forward	5′-TTGTGGCTTGCTAATAAT-3′	[39]
	Reverse	5′-AAACCCCATCCTTTATG-3′	

### Immunofluorescence experiments

Immunofluorescence (IF) experiments were performed according to previously described protocols [7,35,37]. Primary and secondary antibodies used for IF are listed in the Tables 3 and 4, respectively. Immunofluorescence signals were detected by an Axio Observer Z1 inverted microscope and LSM710 Confocal microscope from Carl Zeiss. Images were obtained with AxioVision 4.8 (Carl Zeiss Canada Ltd. Ontario, Toronto, Canada) and Zen 2009 software and assembled using Adobe Photoshop CS5 and Adobe Illustrator CS5. For quantification analysis in neurospheres, three neurospheres were randomly selected and all of the cells within each neurosphere were counted based on 4',6-diamidino-2-phenylindole (DAPI) staining. For cell quantification of differentiating cells at D2 and D8, 8 to 10 random fields were selected under the microscope. Approximately 250 cells from the D2 population and 750 cells from the D8 population were counted based on DAPI labeling. The cell counting was done using the ImageJ program.

**Table 3:** Primary antibodies used

**Primary antibody**	**Application and dilution**	**Description**	**Source**
MeCP2 (C-terminal)	IF 1:200	Rabbit polyclonal	Millipore, Billerica, MA, USA, 07-013
MeCP2 (C-terminal)	WB 1:100	Mouse monoclonal	Abcam, Ontario, Toronto, Canada, Ab50005
	IF 1:200		
MeCP2E1	WB 1:100	Chicken polyclonal	Custom-made [7]
GFAP	IF 1:200	Mouse monoclonal	Invitrogen, Life Technologies Inc, Burlington, Ontario, Canada 421262
TUBULIN III (TUB III)	IF 1:200	Mouse monoclonal	Chemicon, Millipore, Billerica, MA, USA MAB1637
OLIG2	IF 1:200	Rabbit polyclonal	Millipore, Millipore, Billerica, MA, USA, AB9610
NESTIN	IF 1:230	Rat monoclonal	Developmental Studies Hybridoma Bank, Rat-401c
S100B	IF 1:100	Mouse monoclonal	Abcam, Ontario, Toronto, Canada, ab4066
CNPase	IF 1:100	Mouse monoclonal	Covance, SMI-91R
MBP	IF 1:100	Rabbit polyclonal	Abcam, Ontario, Toronto, Canada, ab40390
NEUN	IF 1:200	Mouse monoclonal	Millipore, Billerica, MA, USA, Mab377
KI67	IF 1:200	Rabbit polyclonal	Santa cruz, Dallas, Texas, USA, sc-15402
5mC	Dot blot 1:1,000	Mouse monoclonal	Abcam, Ontario, Toronto, Canada, Ab73938
	IF 1:200		
5hmC	Dot blot 1:1,000	Rabbit polyclonal	Active Motif, 39769

MeCP2, Methyl CpG binding protein; GFAP, Glial fibrillary acidic protein; OLIG2, Oligodendrocyte lineage transcription factor 2; CNPase, 2',3'-Cyclic-nucleotide 3'-phosphodiesterase, MBP, Myelin basic protein; NEUN, NEUronal Nuclei; 5mC, 5-methylcytosine; 5hmc, 5-hydroxymethylcytosine; IF, Immunofluorescence; WB, Western blot.

**Table 4:** Secondary antibodies used

**Secondary antibody**	**Application and dilution**	**Source**
FITC conjugated goat anti rabbit IgG	IF 1:400	Jackson Immunoresearch, PA, USA, 111-095-144
Rhodamine Red-X conjugated goat anti mouse IgG	IF 1:400	Jackson Immunoresearch, PA, USA, 115-259-146
Dylight 649 conjugated goat anti chicken IgY	IF 1:400	Jackson Immunoresearch, PA, USA, 103-485-155
Dylight 649 conjugated donkey anti goat IgG	IF 1:400	Jackson Immunoresearch, PA, USA, 705-494-147
Alexa Fluor 594 conjugated donkey anti mouse IgG	IF 1:1,000	Life Technologies Inc, Ontario, Canada, 987237
Alexa Fluor 448 conjugated donkey anti rabbit IgG	IF 1:1,000	Life Technologies Inc, Ontario, Canada, 913921
Peroxidase-Affinipure Gt anti-mouse IgG	WB 1:7,500	Jackson ImmunoResearch, PA, USA, 115-035-174
	Dot blot 1:7,500	

IF, Immunofluorescence; WB, Western blot.

### Nuclear extractions and western blotting

Nuclear extraction from D2 and D8 NSC were carried out using the NE-PER Nuclear and Cytoplasmic Extraction Kit (Thermo Scientific, Ontario, Toronto, Canada), as per the manufacturer’s instructions. Western blot (WB) experiments were conducted according to previously described protocols [49-51], and quantification of the signals was performed as reported [7]. ACTIN or glyceraldehyde-3-phosphate dehydrogenase (GAPDH) was used as a loading control. Student *t*-test was used to determine statistical significance between control and treated cells. Primary and secondary antibodies used for WB are listed in the Tables 3 and 4, respectively.

### DNA dot blot assay for 5mC and 5hmC

Genomic DNA was isolated using the DNeasy Blood and Tissue kit (Qiagen, Ontario, Toronto, Canada). DNA dot blot was performed using a previously described protocol [52], with minor modifications. The DNA blotted membranes were probed with either 5mC or 5hmC antibody (Tables 3 and 4). Total DNA levels were detected by staining the same membrane with 0.02% methylene blue (MB) in 0.3 μM sodium acetate (pH 5.2). Adobe Photoshop CS5 software was used to quantify dot blot signals.

### Bisulfite pyrosequencing

Genomic DNA was isolated as described in the previous section. Primer design and bisulfite pyrosequencing experiments were conducted as a service by The Hospital for Sick Children (SickKids), Toronto, Canada, as reported elsewhere [53], using the primers listed in Table 5. The regions analyzed for the methylation patterns are shown in Figure 1B. Specific CpG dinucleotides that are analyzed within each region are shown in Additional file 1.

**Table 5:** Primers used in bisulfite pyrosequencing

***Mecp2 *** **region**	**Sequence**
**Region 1**	F: 5′-TGGGTTTTATAATTAATGAAGGGTAA-3′
	R: 5′-CGCCAGGGTTTTCCCAGTCACGACATTTTACCACAACCCTCTCT-3′
	S: 5′-AGGTGTAGTAGTATATAGG-3′
**Region 2**	F: 5′-AGTTTGGGTTTTATAATTAATGAAGGG-3′
	R: 5′-CGCCAGGGTTTTCCCAGTCACGACATTTTACCACAACCCTCTCT-3′
	S: 5′-AAGGGTAATTTAGATAAAGAGTAAG-3′
**Region 3**	F: 5′-GGTGAATTATTTAGTAGGGAGGTTTTAA -3′
	R: 5′-CGCCAGGGTTTTCCCAGTCACGACAAAAAAAAAACCAACCCCATTCAACTAC -3′
	S: 5′-AGTAGGGAGGTTTTAATAG -3′
**Region 4**	F: 5’-GTTTTAAAAAGTTTTGGGAAAAGGTGTAGT -3′
	R: 5′-CGCCAGGGTTTTCCCAGTCACGACCTAAACCCTAACATCCCAACTACCAT-3′
	S: 5′-AGTTTAATGGGGATTTTTAATT -3′
**Region 5**	F: 5′-AGTAGAAGTTATTATTTGTGGTGTGTAT -3′
	R: 5′-CGCCAGGGTTTTCCCAGTCACGACACTATATTACTTCCCAACTCAACTAATT -3′
	S: 5′-AGAGGTGTAAGGATTTT -3′
**Region 6**	F: 5′-GAAGTAGGAAGAATTGAGTTTGAGGATAG -3′
	R: 5′-CGCCAGGGTTTTCCCAGTCACGACATCTATACACTACCCACATATAATACC -3′
	S: 5′-GTTTGAGGATAGTTTGAAT -3′

F: Forward PCR primer, R: Reverse PCR primer (Biotinylated), S: Sequencing primer.

### Correlation analysis

The correlation between DNA methylation at the *Mecp2* REs and expression of *Mecp2* isoforms was determined using the Pearson’s correlation analysis and linear regression. The Pearson’s correlation coefficient (*r*) was calculated for average methylation against each *Mecp2* isoform, over entire regions and for individual CpG sites within each region. The strength of correlation was considered as follows: weak, 0 < *r* <0.3; moderate, 0.3 < *r* <0.4; strong, 0.4 < *r* <0.7; or very strong, 0.7 < *r* <1.0. A negative *r*-value indicates an inverse/negative correlation whereas a positive *r*-value indicates direct/positive correlation. Statistical significance was determined at *P* <0.05.

## Results

### Dynamic expression of *Mecp2* isoforms during NSC differentiation and DNA methylation patterns at the *Mecp2* regulatory elements

**Figure 2:**
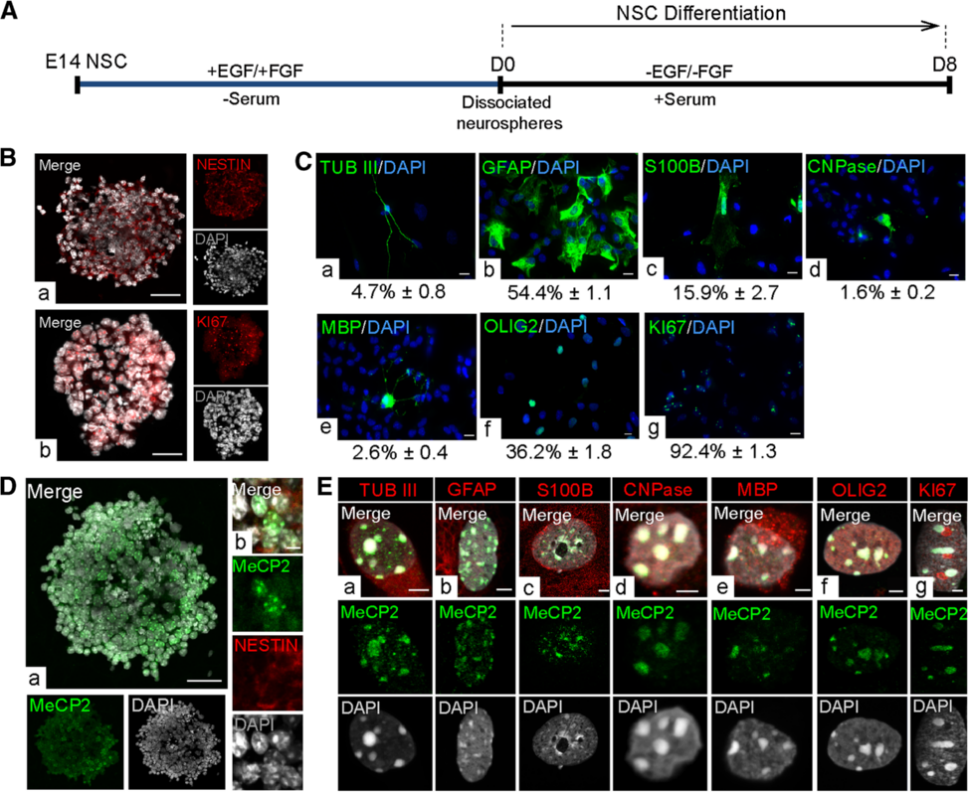
**Characterizing *in vitro *neural stem cells to study Methyl CpG binding protein 2 (MeCP2) expression. (A)** Schematic representation of *in vitro* neural stem cell (NSC) differentiation. **(B)** Detection of (a) NESTIN^+^ and (b) KI67^+^ cells in self-renewing neurospheres. Scale bars represent 20 μm. **(C)** Immunofluorescent detection of different cell-type markers in the day 8 (D8) population (a) TUBULIN III (TUB III): neurons, (b) Glial fibrillary acidic protein (GFAP): astrocytes, (c) S100B: mature astrocytes, (d) 2',3'-Cyclic-nucleotide 3'-phosphodiesterase (CNPase): oligodendrocytes, (e) Myelin basic protein (MBP): oligodendrocytes, (f) Oligodendrocyte lineage transcription factor 2 (OLIG2): early oligodendrocytes and progenitors, and (g) KI67: proliferating cells. Scale bars represent 10 μm. The percentages represent average number of cells from three individual experiments (n = 3 ± standard error of the mean). **(D)** (a) Immunofluorescent detection of MeCP2 in a sectioned primary neurosphere. Scale bar represents 20 μm. (b) Double labeling of MeCP2 and NESTIN within primary neurosphere cells. Scale bar represents 5 μm. **(E)** Immunofluorescent detection of MeCP2 in D8 cell types: (a) TUB III, (b) GFAP, (c) S100B, (d) CNPase, (e) MBP, (f) OLIG2, and (g) KI67. Scale bars represent 2 μm.

Primary neural stem cells were isolated from the embryonic forebrain at E14.5 and were cultured in the presence of growth factors to generate primary neurospheres. After 7 days in culture, primary neurospheres were dissociated and cultured under differentiation conditions for 8 days, reported to be sufficient for differentiation of both neuronal and glial cells [35,37] (Figure 2A). In agreement with our previous report [37] the proliferating primary neurospheres expressed NESTIN and KI67 (Figure 2B). Similar to our previous reports [35,37] differentiated NSC at D8 consisted of a mixed population of neurons, astrocytes, and oligodendrocytes (Figure 2C). The composition of the D8 population was determined by detection of cell type-specific markers (TUB III (4.7% ± 0.8 mean ± standard error of the mean (SEM)), Glial fibrillary acidic protein (GFAP) (54.4% ± 1.1), S100B (15.9% ± 2.7), 2',3'-Cyclic-nucleotide 3'-phosphodiesterase (CNPase) (1.6% ± 0.2), Myelin basic protein (MBP) (2.6% ± 0.42), Oligodendrocyte lineage transcription factor 2 (OLIG2) (36.2% ± 1.8) and KI67 (92.4% ± 1.3)) by immunofluorescence (Figure 2C). KI67 is not a cell type-specific marker, but rather reflects the fraction of cycling cells within differentiating NSC. Indicating that the cells at an early stage of differentiation are actively dividing, expression of KI67 (98.8% ± 0.8) was also detected in D2 cells (Additional file 2). Our detection of KI67 in the majority of cells at D8 indicates that although most differentiating NSC are actively dividing, fewer than 10% of cells are post-mitotic and may include neurons (TUB III^+^) or non-proliferating cells committed towards neuronal cell fate. Taken together, this *in vitro* NSC differentiation system provided a suitable model system consisting of the three main neural cell types (neurons, astrocytes and oligodendrocytes) in the brain to study *Mecp2*/MeCP2 isoforms during neural differentiation.

First, we confirmed MeCP2 expression in neurospheres at D0 and differentiated NSC at D8 by IF studies. We used an antibody that was raised against the MeCP2 C-terminus and recognizes both isoforms. Characteristic punctate nuclear expression of MeCP2 was detected in 41% of neurosphere cells (Figure 2D, a), which were positive for the NSC marker NESTIN (Figure 2D, b). At D8 of NSC differentiation, MeCP2 protein was detected in all cell types in the D8 differentiated progenies, including neurons, astrocytes, and oligodendrocytes (Figure 2E). Indicating the expression of MeCP2 in proliferating cells, we detected MeCP2 in KI67^+^ cells in the D8 population (Figure 2E, g). The detected nuclear MeCP2 signals were enriched at the heterochromatin-rich regions of all three cell types. These observations are consistent with our previous reports on MeCP2 nuclear expression in *in vivo* differentiated primary neurons and astrocytes [7].

**Figure 3:**
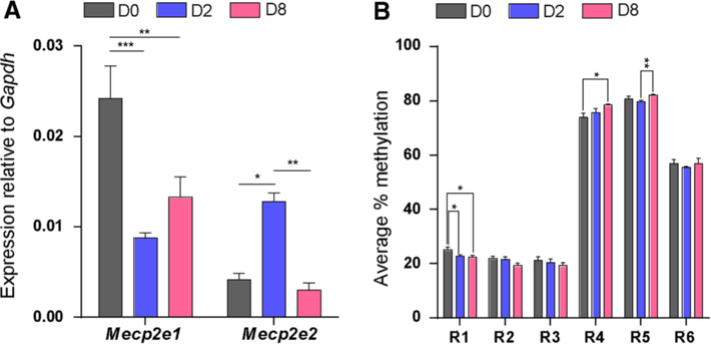
**Methyl CpG binding protein 2 gene (*Mecp2*) isoform-specific transcript expression and DNA methylation at the *Mecp2 *regulatory elements during neural stem cell (NSC) differentiation. (A)** Analysis of *Mecp2e1* and *Mecp2e2* transcript levels during NSC differentiation: n = 3 ± standard error of the mean (SEM). Significant differences: ^***^P <0.001; ^**^P <0.01; ^*^P <0.05. **(B)** Average percentage methylation over *Mecp2* promoter and intron 1 regions at day 0 (D0), day 2 (D2) and day 8 (D8) during NSC differentiation. The regions are promoter regions R1, CpG island contains 13 CpG sites; R2, 4 CpG sites; R3, 2 CpG sites, and intron 1 regions R4, 1 CpG site; R5, 1 CpG site; and R6, 2 CpG sites; n = 3 ± SEM. Significant differences: ^**^P <0.01; ^*^P <0.05. *Gapdh*, glyceraldehyde-3-phosphate dehydrogenase gene.

Next, we investigated *Mecp2* isoform-specific transcript expression at three stages of NSC differentiation: undifferentiated cells (D0), cells at an early stage of differentiation (D2), and cells at a later stage of differentiation (D8). Distinct and mirror-like (reciprocal) transcript expression patterns for *Mecp2* isoforms were observed during NSC differentiation (D0, D2, and D8) (Figure 3A), suggesting possible differential regulation of these isoforms during NSC differentiation. Expression of *Mecp2e1* was reduced from D0 to D2 (2.7-fold, *P* <0.001) and was slightly elevated from D2 to D8. In contrast, *Mecp2e2* expression increased from D0 to D2 (3.1-fold, *P* <0.05), but declined from D2 to D8 (4.2-fold, *P* <0.01). At each of these studied differentiation stages, the expression ratio between the two isoforms (*Mecp2e1/Mecp2e2*) varied significantly (D0, 5.99; D2, 0.69; D8, 4.62) (Additional file 3). At D0 and D8, *Mecp2e1* expression was significantly higher than *Mecp2e2* (D0, *P* <0.01, and D8, *P* <0.05). In contrast at D2, *Mecp2e2* expression was significantly higher than *Mecp2e1* (D2, *P* <0.05). These observations imply differential regulation of *Mecp2* isoforms and possible changes in alternative splicing of *Mecp2* at different stages of NSC differentiation.

*MECP2*/*Mecp2* expression is known to be regulated by REs found within its promoter and intron 1. The altered MeCP2 expression in autistic patients and in mouse brains subjected to stress is correlated with increased *MECP2/Mecp2* promoter methylation [2,12,13,18-20]. Therefore, we hypothesized that DNA methylation at the *Mecp2* promoter and intron 1 might impact *Mecp2* isoform-specific expression. For DNA methylation analysis by bisulfite pyrosequencing, we selected three regions within the *Mecp2* promoter (named R1 to R3, hereafter) and three regions within the intron 1 (named R4 to R6, hereafter) (Figure 1B). These regions harbored different numbers of CpG sites; promoter regions R1: CpG island contains 13 CpG sites; R2: 4 CpG sites; R3: 2 CpG sites, and intron 1 regions R4: 1 CpG site; R5: 1 CpG site; and R6: 2 CpG sites.

Pyrosequencing analysis of R1-R6 indicated that downregulation of *Mecp2e1* and upregulation of *Mecp2e2* from D0 to D2 were associated with slight, but significant demethylation of *Mecp2* promoter R1 (2.3%, *P* <0.05). Similarly, upregulation of *Mecp2e1* and downregulation of *Mecp2e2* from D2 to D8 were associated with hypermethylation of *Mecp2* intron 1 R5 (2.4%, *P* <0.01) (Figure 3B). Detected expression changes in *Mecp2* isoforms from D0 to D8 were associated with demethylation of *Mecp2* promoter R1 (2.6%, *P* <0.05), and hypermethylation of *Mecp2* intron 1 R4 (4.6%, *P* <0.05). In all cases, the differences in average percentage methylation between D0, D2 and D8 were relatively small, but statistically significant and ranging between 2 to 5%. Previous reports have shown that an increase in the overall *MECP2* promoter methylation by approximately 2.0 to 2.5% in male autistic patients correlates with significantly reduced *MECP2* expression levels [2]. In mouse brain exposed to maternal separation and stress, DNA methylation changes that are as little as 2 to 5% at individual CpG sites of the *Mecp2* promoter are associated with significantly reduced MeCP2 expression [20]. As even slightly altered *MECP2/Mecp2* promoter methylation (2 to 5%) affects *MECP/Mecp2* gene expression in the human and mouse brain, it is likely that the statistically significant changes detected in the present study might be biologically important for *Mecp2e1* and/or *Mecp2e2* expression.

As mentioned, the ratio of *Mecp2* splice variants was changed at different stages of NSC differentiation. Therefore, we performed Pearson's correlation analysis between *Mecp2e1/Mecp2e2* expression ratio and DNA methylation at R1, R4 and R5 (the three regions that showed significant changes) during NSC differentiation. Pearson’s correlation coefficient (*r*) represents the strength of correlation, with negative *r* indicating inverse correlation, and positive *r* indicating direct correlation between DNA methylation and the *Mecp2e1/Mecp2e2* splice ratio. We detected a statistically significant positive correlation (*r* >0.9, *P* <0.01) between *Mecp2e1/Mecp2e2* splice ratio at D2 and DNA methylation at intron 1 R4 (Additional file 3). Although it is possible that intron 1 (R4) may play a role in alternative splicing of *Mecp2*, further investigations are required to establish the involvement of DNA methylation in *Mecp2* transcriptional splicing.

As *MECP2*/*Mecp2* is an X-linked gene, it is possible that the observed changes in *Mecp2* expression are due to a shift in the number of cells derived from male and female embryos. To exclude such a possibility, we determined the contribution from the male/female embryonic cells during NSC differentiation using a semiquantitative PCR-based method. Genomic DNA was extracted from each differentiation stage and subjected to PCR analysis for the presence of the *Sry* gene found in the Y chromosome. The autosomal gene *Il3* was used an internal control. The adult male brain cortex was used as a positive control for the presence of the male genomic DNA (Additional file 4: Figure S4A). We did not observe statistically significant changes in the ratio of *Sry/Il3* in the cells collected at different stages of differentiation, indicating that the ratio of male/female differentiating NSC were unchanged (Additional file 4: Figure S4B). To further confirm the contribution from the male/female gender, we tested the transcript levels of *Xist* gene (the gene is involved in X-chromosome inactivation) by qRT-PCR. We did not detect any significant change in *Xist* gene expression at different stages of NSC differentiation (Additional file 4: Figure S4C). These results indicate that our observed changes in *Mecp2* expression are not due to altered contribution of male and female cells. Taken together, our results suggest a possible link between the *Mecp2* isoform-specific expression and DNA methylation at the *Mecp2* REs within the *Mecp2* promoter and intron 1 during NSC differentiation.

### Decitabine exposure leads to *Mecp2e1* upregulation but its withdrawal downregulates both *Mecp2* isoforms to different extents

**Figure 4:**
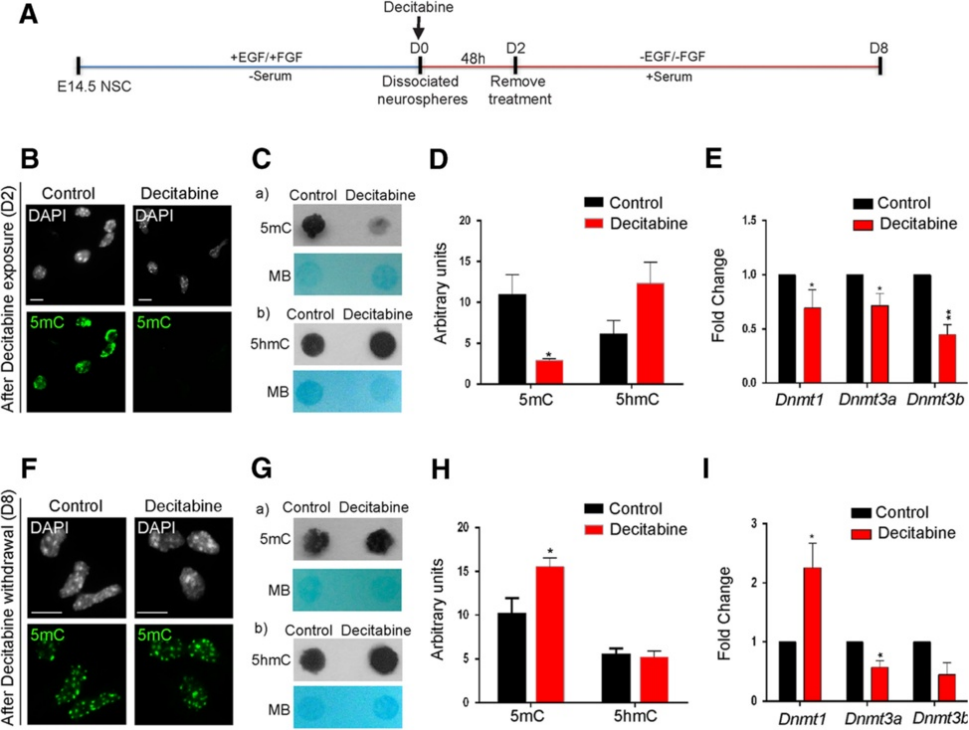
**Effect of decitabine on global DNA methylation (5mC and 5hmC) and *Dnmt *genes in differentiating neural stem cells (NSC). (A**) Schematic representation of decitabine treatment. Briefly, 2.5 μM of decitabine was added to dissociated neurospheres on day 0 (D0) at the onset of NSC differentiation for 48 h, and the treatment was withdrawn at D2. Cells were kept in culture till D8. Top panel **(B**-**E)**, after exposure to decitabine at D2. **(B)** Immunofluorescent detection of DNA methylation using 5-methylcytosine (5mC) antibody. Decitabine caused reduced levels of DNA methylation, note the presence of 4',6-diamidino-2-phenylindole (DAPI) signals in decitabine-treated cells with no 5mC signal. Scale bars represent 5 μm. **(C)** Detection of overall DNA methylation levels by DNA dot blot with antibodies specific for (a) 5mC, (b) 5-hydroxymethylcytosine (5hmC). **(D)** Quantification of the 5mC and 5hmC levels after decitabine exposure. **(E)** Detection of *Dnmt* transcript levels by qRT-PCR. Bottom panel **(F-I)**, after withdrawal of decitabine at D8. **(F)** DNA methylation detection by immunofluorescence using 5mC antibody. Scale bars represent 5 μm. **(G)** Detection of global DNA methylation levels by DNA dot blot, (a) 5mC, (b) 5hmC. **(H)** Quantification of 5mC and 5hmC levels after withdrawal of decitabine. **(I)** Detection of *Dnmt* transcript levels by qRT-PCR. Fold changes are calculated relative to transcript levels at D2 or D8 control; n = 3 ± standard error of the mean. Significant differences from control: ^**^P <0.01; ^*^P <0.05. MB, methylene blue (used for visualizing total DNA).

To further study the impact of DNA demethylation/remethylation in *Mecp2* isoform-specific expression, we treated dissociated neurosphere cells with 2.5 μM decitabine for 48 h, at the onset of NSC differentiation (D0) (Figure 4A). At D2, decitabine was withdrawn from the media and cells were kept in culture for another 6 days until D8, to study the effect of DNA remethylation (Figure 4A). First, as a proof of principle, we verified whether decitabine acted as a DNA demethylating agent in our system. Global change in DNA methylation was determined by IF for 5mC and DNA dot blot assay for both 5mC and 5hmC. As expected, IF experiments showed that 5mC nuclear signals were noticeably lower in decitabine-treated NSC compared to D2 control untreated cells (Figure 4B). DNA dot blot assays indicated that decitabine treatment resulted in reduced 5mC levels (3.79-fold, *P* <0.05), with slight but statistically insignificant increase in 5hmC levels (Figure 4C-D). In contrast, decitabine withdrawal led to re-establishment of global DNA methylation (5mC) at D8 as detected by IF (Figure 4F). Furthermore, DNA dot blot assay showed DNA methylation reprogramming upon decitabine withdrawal, with elevated 5mC levels (1.5-fold, *P* <0.05), and relatively unchanged 5hmC levels in decitabine-treated NSC compared to controls (Figure 4G-H). Although globally altered 5mC levels were expected following decitabine treatment in agreement with previous studies [54,55], the observed effect of decitabine to slightly increase 5hmC levels was novel and might be biologically important.

DNA demethylating agents can function as cytosine analogues and/or as *Dnmt*/DNMT inhibitors [56]. Therefore, we investigated *Dnmt* expression levels in decitabine-treated differentiating NSC by qRT-PCR. In accordance with reduced DNA methylation levels at D2, decitabine treatment caused significant inhibition of transcript levels of all three DNA methyltransferases (*Dnmt1*, 1.7-fold, *P* <0.05; *Dnmt3a*, 1.5-fold, *P* <0.05 and *Dnmt3b*, 2.5-fold, *P* <0.01) (Figure 4E). Even though we anticipated that decitabine withdrawal would restore *Dnmt* levels, only *Dnmt1* levels were elevated (2.2-fold, *P* <0.05), whereas both *Dnmt3a* (1.4-fold, *P* <0.05) and *Dnmt3b* (1.8-fold, *P* = 0.06) levels remained inhibited (Figure 4I). In summary, these results indicate that decitabine functions as a DNA demethylating agent in differentiating NSC and globally affects DNA methyl marks. Additionally, our data indicate that decitabine withdrawal would lead to DNA methylation reprogramming in differentiating NSC.

**Figure 5:**
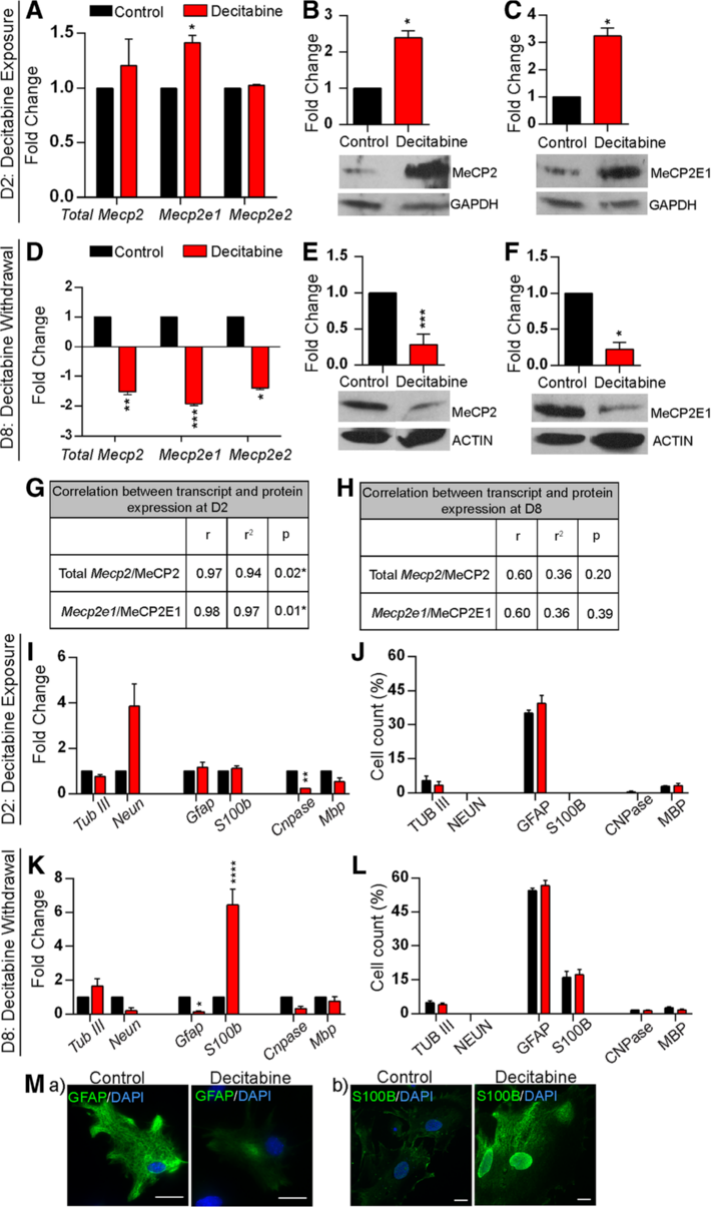
**Effect of decitabine exposure and withdrawal on Methyl CpG binding protein gene (*Mecp2*/MeCP2) expression. (A-C)**, After exposure to decitabine at D2. **(A)** Analysis of *Mecp2* (total), *Mecp2e1* and *Mecp2e2* transcript levels by qRT-PCR. **(B)** Detection of MeCP2 (total) protein expression levels by western blot; n = 2 ± standard error of the mean (SEM). **(C)** Detection of MeCP2E1 protein expression levels by western blot; n = 2 ± SEM. **(D-F)**, After withdrawal of decitabine at day 8 (D8). **(D)** Analysis of total *Mecp2*, *Mecp2e1* and *Mecp2e2* transcript levels by qRT-PCR. **(E)** Detection of MeCP2 (total) protein expression levels by western blot. **(F)** Detection of MeCP2E1 protein expression levels by western blot. **(G-H)** Pearson's correlation analysis of the relation between *Mecp2* transcript levels and MeCP2 protein levels at D2 **(G)** and D8 **(H)**; *r* = Pearson’s correlation coefficient, *r*^2^ = coefficient of determination. **(I)** Transcript detection of cell type-specific markers for neurons (*Tub III*, *NeuN*); astrocytes (*Gfap*, *S100b*); oligodendrocytes (*Cnpase*, *Mbp*) by qRT-PCR in D2 control and decitabine-treated cells. **(J)** Quantification of neurons, astrocytes and oligodendrocytes using cell type-specific markers by immunofluorescence in D2 control and decitabine-treated cells. **(K)** Transcript detection of cell type-specific markers for neurons (*Tub III*, *NeuN*); astrocytes (*Gfap*, *S100b*); oligodendrocytes (*Cnpase*, *Mbp*) by qRT-PCR in D8 control and decitabine-treated cells. **(L)** Quantification of neurons, astrocytes and oligodendrocytes using cell type-specific markers in D8 control and decitabine-treated cells. **(M)** Comparison of immunofluorescent detection of (a) Glial fibrillary acidic protein (GFAP) and (b) S100B between control and decitabine-treated cells. Images were taken at the same exposure time. Scale bars represent 20 μm. For all the panels, fold changes are calculated relative to expression levels at D2 or D8 controls. Significant differences from controls: ^****^P <0.0001; ^***^P <0.001; ^**^P <0.01; ^*^P <0.05, n = 3 ± SEM, unless specifically mentioned.

Next, we investigated possible changes in *Mecp2*/MeCP2 expression induced by decitabine. Quantitative RT-PCR experiments indicated that decitabine treatment at D2 caused slight but statistically significant upregulation of *Mecp2e1* (1.41-fold, *P* <0.05), with minimal and insignificant increased levels of the total *Mecp2* (1.2-fold, *P* = 0.5), and unchanged levels of *Mecp2e2* (Figure 5A). Analysis of protein levels by WB showed that decitabine upregulated total MeCP2 (2.5-fold, *P* <0.05), and MeCP2E1 (3.1-fold, *P* <0.05) protein expression (Figure 5B-C). However, the lack of an antibody specific for MeCP2E2 limited our investigation of MeCP2E2 protein levels. Correlation analysis of transcript and protein levels of *Mecp2*/MeCP2 at D2 indicated significant correlation between the detected transcript and protein expression (*Mecp2*/MeCP2 (*r* = 0.97, *P* <0.05) and *Mecp2e1*/MeCP2E1 (*r* = 0.98, *P* <0.01); Figure 5G).

In contrast to D2, withdrawal of decitabine at D8 significantly downregulated the transcript expression levels of *Mecp2e1* (1.92-fold, *P* <0.001), *Mecp2e2* (1.39-fold*, P* <0.05) and the total *Mecp2* (1.52-fold, *P* <0.01) (Figure 5D). Similar to the transcript levels, decitabine withdrawal resulted in downregulation of total MeCP2 (3.2-fold, *P* <0.001), and MeCP2E1 (4.3-fold, *P* <0.05) protein expression levels (Figure 5E-F). A similar correlation analysis between transcript and protein levels of *Mecp2*/MeCP2 at D8 did not show any statistically significant correlation (*Mecp2*/MeCP2 (*r* = 0.6, *P* = 0.2), and *Mecp2e1*/MeCP2E1 (*r* = 0.6, *P* = 0.3); Figure 5H). These observations emphasize that even minor change in *Mecp2* transcript levels are biologically important and can result in significantly altered MeCP2 protein expression levels.

Next, we aimed to study whether the detected changes in *Mecp2*/MeCP2 expression were due to changes in cell population in response to decitabine treatment. Therefore, we studied the effect of decitabine on cell fate commitment of differentiating NSC at D2 and D8. After decitabine exposure at D2, we examined the expression of cell type-specific markers (neurons: *Tub III*, *NeuN*; astrocytes: *Gfap*, *S100b*; oligodendrocytes: *Cnpase*, *Mbp*) at the transcript levels by qRT-PCR. Comparing the control and decitabine-treated cells, we did not detect any statistically significant change in these cell type-specific genes, except for significant downregulation of *Cnpase* (9-fold, *P* <0.01) (Figure 5I). In order to determine whether any of these detected changes in transcript levels are represented in the number of cells expressing each corresponding cell type-specific marker, we performed IF experiments with specific antibodies against these markers (Figure 5J). IF experiments showed that there was no significant change in the number of TUB III^+^, GFAP^+^, CNPase^+^, or MBP^+^ cells. However, we did not find any NEUN^+^, or S100B^+^ cells in the control or decitabine-treated populations at D2, probably because these cells are still in the early stages of differentiation (Figure 5J). In the D8 population, decitabine treatment led to insignificant changes in the transcript levels for all neuronal and oligodendrocyte markers compared to control untreated cells. Additionally, *Gfap* expression in decitabine-treated cells was downregulated 5.5-fold, whereas *S100b* expression was upregulated to a similar extent (6-fold) (Figure 5K). Quantification of differentiated neurons, astrocytes and oligodendrocytes at D8 by IF did not show any significant change in the cell-fate commitment of these cells (Figure 5L). However, reduced *Gfap* expression in decitabine-treated cells without any changes in the number of GFAP^+^ cells might be explained by the reduced intensity of GFAP staining relative to the control astrocytes, since the images were taken at the same exposure level (Figure 5M, a). Similarly, the significant upregulation of *S100b* transcript levels by decitabine with no change in the number of S100B^+^ cells could be explained by the increased intensity of S100B in decitabine-treated cells, when the images were taken at the same exposure time (Figure 5M, b). Taken together, these results suggest that decitabine has minimal effect on the differentiated number of neurons, astrocytes and oligodendrocytes under the described conditions. They further suggest that the detected changes in *Mecp2* expression are not likely due to altered population of differentiating cell types.

Next, we investigated whether the observed altered *Mecp2*/MeCP2 expression was due to changes in the number of cells deriving from male and female embryos. Detection of *Sry* and *Il3* by PCR indicated that the ratio of *Sry/Il3* was relatively similar in D2 control and decitabine-treated populations (Additional file 4: Figure S4D). Similar PCR analysis at D8 also showed no significant differences in the ratio of *Sry/Il3* between D8 control and decitabine-treated cells (Additional file 4: Figure S4E). Furthermore, qRT-PCR analysis of the *Xist* gene expression in both D2 and D8 populations with and without decitabine treatment showed no significant change in *Xist* transcript expression levels between the control and decitabine-treated cells (Additional file 4: Figure S4F-G). Therefore, these results indicate that the observed changes in *Mecp2*/MeCP2 expression in response to decitabine exposure and withdrawal are not due to a shift in the number of cells deriving from male/female embryos.

Taken together, our data so far indicate that a single administration of decitabine for 48 h induces *Mecp2e1*/MeCP2E1, MeCP2 (total) expression, whereas its withdrawal downregulates *Mecp2* (total)*/*MeCP2 (total), *Mecp2e1*/MeCP2E1, and *Mecp2e2* expression with minimal change in NSC differentiation.

### Decitabine mediates altered DNA methylation patterns at the *Mecp2* regulatory elements

**Figure 6:**
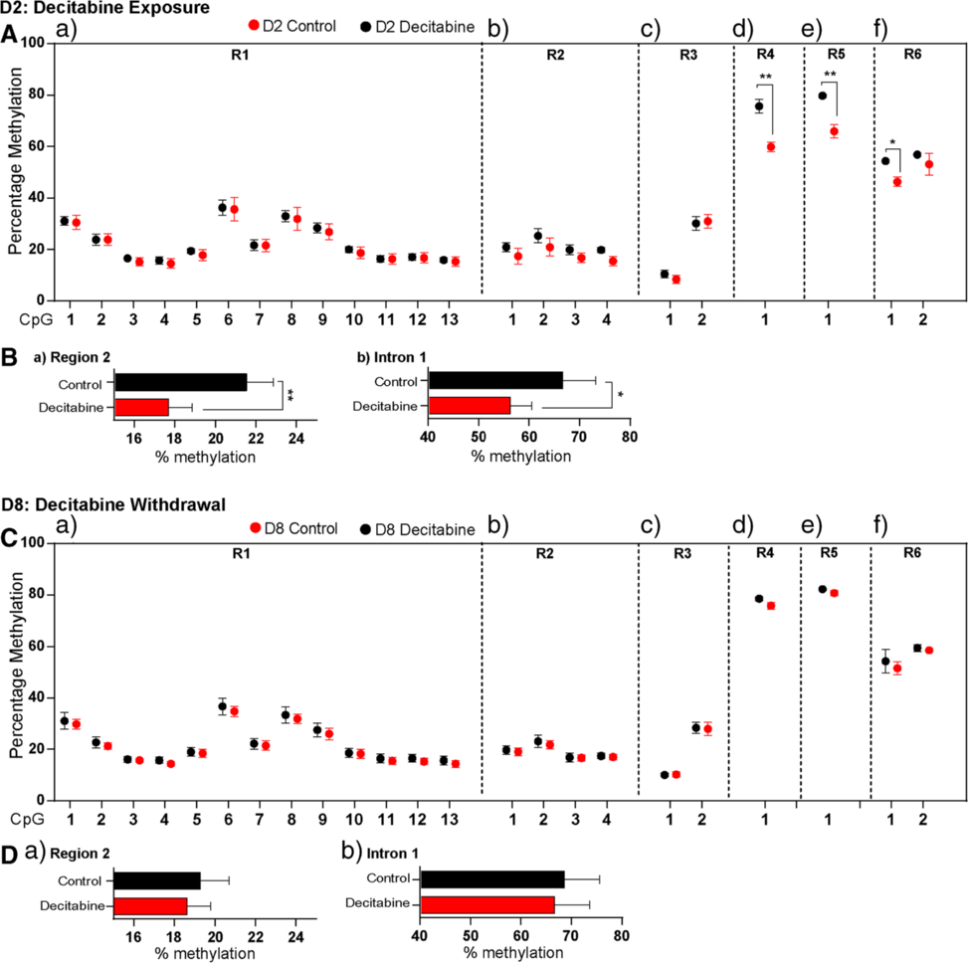
**Bisulfite pyrosequencing analysis of DNA methylation at the Methyl CpG binding protein 2 gene (*Mecp2*) regulatory elements after decitabine treatment. (A)** Effect of decitabine exposure at D2 on the percentage DNA methylation of *Mecp2* regulatory regions. The three promoter regions are (a) R1, (b) R2, (c) R3, and the three intron 1 regions are (d) R4, (e) R5, and (f) R6. **(B)** Effect of decitabine exposure on average methylation over the entire region 2 (R2) (a), and intron 1 (R4 to R6) (b). Significant differences from controls: ^**^P <0.01; ^*^P <0.05; n = 3 ± standard error of the mean. **(C)** Effect of decitabine withdrawal at D8 on percentage methylation of *Mecp2* regulatory regions. The regions are promoter regions (a) R1, (b) R2 and (c) R3, and intron 1 regions (d) R4, (e) R5 and (f) R6. **(D)** Effect of decitabine withdrawal on average DNA methylation over entire region 2 (R2) (a), and intron 1 (R4 to R6) (b).

As mentioned earlier, DNA methylation changes at the overall *MECP2* promoter and individual CpG sites within the *MECP2*/*Mecp2* promoter are associated with altered *MECP2*/*Mecp2* expression [2,19,20]. Therefore, we investigated whether altered expression of *Mecp2* isoforms in our NSC system are associated with change in DNA methylation at the *Mecp2* REs found within the *Mecp2* promoter and intron 1. Bisulfite pyrosequencing analysis showed that decitabine treatment (D2) caused no significant change in the percentage DNA methylation at the *Mecp2* promoter R1 and R3 (Figure 6A, a, c). However, decitabine caused demethylation of all individual CpG dinucleotides at the R2 (CpG1, 3.5%; CpG2, 4.4%; CpG3, 3.1%; CpG4, 4.28%) (Figure 6A, b), as well as the average R2 percentage DNA methylation (3.83%, *P* <0.05) (Figure 6B, a). Similarly, decitabine caused demethylation of individual CpG sites at the intron 1 R4 (15.8%, *P* <0.05), R5 (13.08%, *P* <0.05), and R6 (CpG1, 8.01%, *P* <0.01; CpG2, 3.8%, *P* = 0.4) (Figure 6A, d-f), with significant demethylation at the entire intron 1 (10.37%, *P* <0.05) (Figure 6B, b). These results indicated that decitabine induced significant DNA demethylation at both the *Mecp2* promoter and intron 1, at the individual CpG sites and the overall DNA methylation. As mentioned earlier, this detected DNA demethylation was associated with significant upregulation of *Mecp2e1* isoform, but not *Mecp2e2.* Therefore, it is possible that the observed changes in DNA methylation at the studied REs contribute to the upregulation of *Mecp2e1*.

Similar bisulfite pyrosequencing analysis at D8 indicated that the three *Mecp2* promoter regions (R1 to R3) and intron 1 regions (R4 to R6) were remethylated and DNA methylation was almost re-established following decitabine withdrawal (Figure 6C). Analyzing the average DNA methylation over the *Mecp2* promoter R2 and the entire intron 1 (which were demethylated at D2), we observed no significant differences in DNA methylation between D8 control and decitabine-treated cells (Figure 6D). Despite the fact that DNA remethylation is expected to restore the gene expression levels, expression of both *Mecp2* isoforms were significantly downregulated. This observation implies that at D8, other regulatory mechanisms apart from promoter/intron 1 DNA methylation might be involved in downregulating *Mecp2* expression.

Taken together, these results show that the induced *Mecp2e1* (but not *Mecp2e2*) expression is associated with reduced DNA methylation at the *Mecp2* REs and decreased global 5mC DNA methylation. Hence, our findings implicate the possible role of *Mecp2* gene-specific DNA demethylation at the specific REs on the expression of *Mecp2e1*/MeCP2E1, and MeCP2 (total) at D2. Moreover, altered expression of *Mecp2* isoforms without any change in DNA methylation at the *Mecp2* REs at D8 imply that mechanisms other than DNA methylation could be involved in downregulating *Mecp2* isoforms.

### *Mecp2* isoform-specific expression correlates with DNA methylation at the *Mecp2* regulatory elements

In order to establish a link between *Mecp2* isoform-specific expression and DNA methylation, we performed Pearson's correlation analysis by comparing normalized (log2) expression of *Mecp2* in each dataset to the respective average percentage methylation levels over entire regions, as well as methylation at individual CpG sites (from both control and decitabine-treated cells).

**Figure 7:**
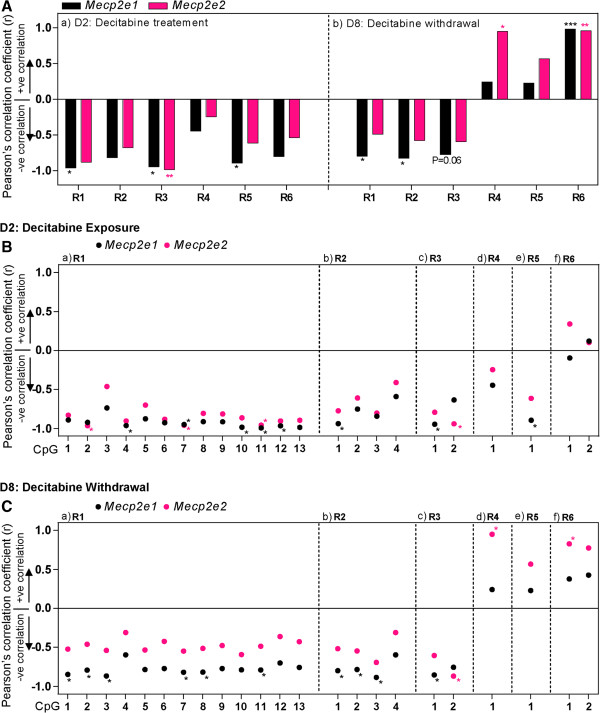
**Correlation analysis between DNA methylation at the Methyl CpG binding protein 2 gene (*Mecp2*) regulatory elements and *Mecp2 *expression after decitabine treatment (day 2) and decitabine withdrawal (day 8).** All graphs represent the Pearson's correlation coefficient (*r*) for *Mecp2e1* (black), and *Mecp2e2* (pink): statistical significance: ^***^P <0.001; ^**^P <0.01; ^*^P <0.05; n = 3. **(A)** Correlation coefficients for the relation between *Mecp2* expression and average methylation over entire regions in *Mecp2* promoter (region (R)1 to R3) and intron 1 (R4 to R6) after decitabine exposure on day 2 (D2) (a), and after decitabine withdrawal on D8 (b). **(B)** After decitabine exposure: correlation coefficients for *Mecp2e1* (black), and *Mecp2e2* (pink) with individual CpG methylation at the promoter regions (a) R1, (b) R2 and (c) R3, and intron 1 regions (d) R4, (e) R5 and (f) R6. **(C)** After decitabine withdrawal: correlation coefficients for *Mecp2e1* (black), and *Mecp2e2* (pink) with individual CpG methylation at promoter regions (a) R1, (b) R2 and (c) R3, and intron 1 regions (d) R4, (e) R5 and (f) R6. Statistical significance: ^*^P <0.05; n = 3.

First, we tested whether average DNA methylation of the entire *Mecp2* promoter (R1 to R3), and intron 1 (R4 to R6) regions correlate with *Mecp2e1* and *Mecp2e2* expression at D2 in control and decitabine-treated cells. We observed a significant negative correlation between *Mecp2e1* expression and the average methylation at R1, R3 and R5 (*r* > -0.9, *P* <0.05). Correlation between *Mecp2e1* upregulation and significant demethylation at R5, induced by decitabine at D2, suggests a possible contribution of R5 in upregulating *Mecp2e1*. On the other hand, *Mecp2e2* showed significant negative correlation only with R3 methylation (*r* > -0.9, *P* <0.05) (Figure 7A, a), that remained unchanged at D2, and this could explain the unaffected *Mecp2e2* expression at D2.

Similar correlation analysis at D8 (in control D8 and decitabine-treated cells), indicated that *Mecp2e1* shows a significant negative correlation with average DNA methylation at the promoter R1 (*r* > -0.7, *P* <0.05), R2 (*r* > -0.8, *P* <0.05) and R3 (*r* > -0.7, *P* = 0.06, close to significant) and a significant positive correlation with the average DNA methylation at the intron 1 R6 (*r* >0.9, *P* <0.001) (Figure 7A, b). In contrast, *Mecp2e2* did not show any significant correlation with any of the promoter regions (R1 to R3), but showed a significant positive correlation with the average methylation at intron 1 R4 (*r* >0.9, *P* <0.05) and R6 (*r* >0.9, *P* <0.01). This divergence in the correlation patterns (negative and positive depending on the stage of differentiation), might imply a potential dynamic role of DNA methylation in regulating *Mecp2* isoforms at different stages of NSC differentiation.

**Figure 8:**
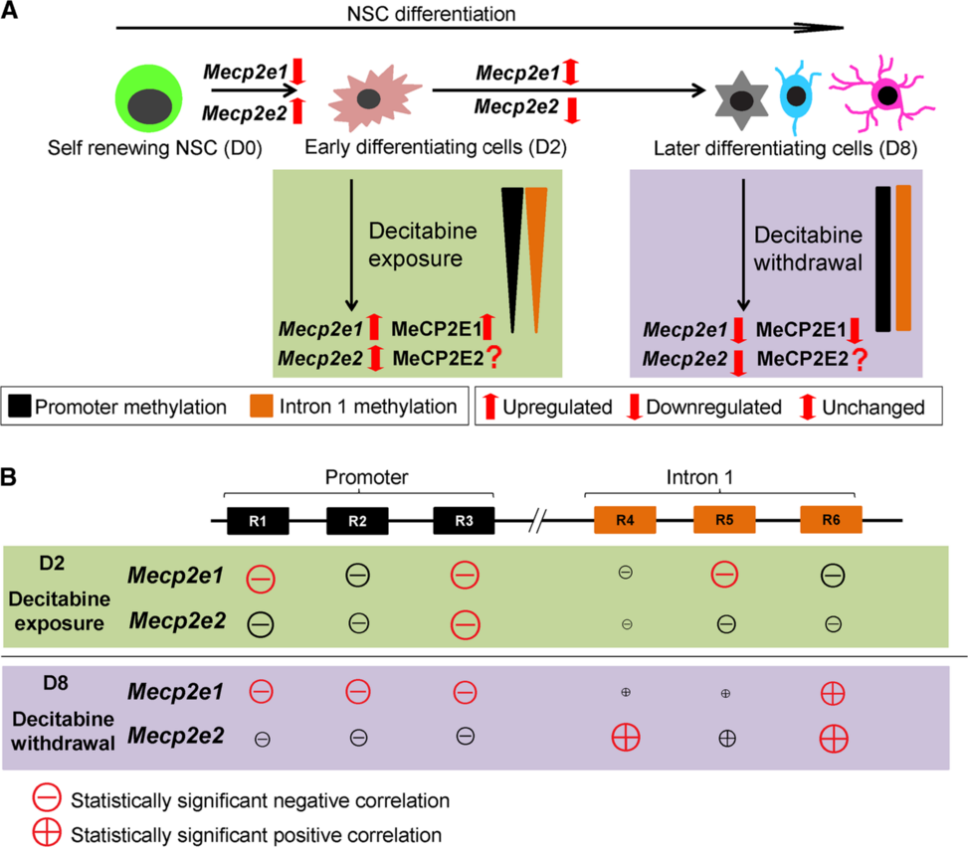
**Summary of the correlations between the expression of Methyl CpG binding protein 2 gene (*Mecp2*) isoforms and DNA methylation at the *Mecp2 *regulatory elements. (A)** Dynamic changes in the expression of *Mecp2* isoforms (*Mecp2e1* and *Mecp2e2*) at different time points of neural stem cell (NSC) differentiation at day 0 (D0), D2 and D8. Decitabine caused upregulation of *Mecp2e1*/MeCP2E1 but not *Mecp2e2* at D2. Decitabine effect on MeCP2E2 at the protein levels is unknown. Decitabine withdrawal by D8 downregulated *Mecp2e1*/MeCP2E1, and *Mecp2e2/*MeCP2E2 (unknown) to different extents. **(B)** Schematic representation of the correlation between *Mecp2* isoform-specific expression and DNA methylation at the *Mecp2* promoter regions (R1 to R3), and intron 1 regions (R4 to R6). The size of the signs, plus (+), and minus (-) represents the relative degree of correlation with either *Mecp2* isoform. The statistically significant correlations are represented in red (red-circled plus, red-circled minus). After decitabine exposure *Mecp2e1* expression negatively correlated with promoter R1 and R3, and intron 1 R5. *Mecp2e2* isoform negatively correlates with promoter R3. In contrast, after decitabine withdrawal, *Mecp2e1* expression negatively correlated with promoter R1, R2 and R3, and positively correlated with intron 1 R6. Hence, correlation between *Mecp2e1* and DNA methylation at REs changed depending on the stage of NSC differentiation. *Mecp2e2* isoform positively correlated with intron 1 R4 and R6.

Last, we investigated whether individual CpG sites within the studied regions (R1 to R6) showed specific correlation with either *Mecp2* isoform (Figure 7B-C). Implicating the possible role of promoter R1 and R2 in mainly regulating *Mecp2e1* (major isoform) at both D2 and D8, we observed a negative correlation between CpG methylation and *Mecp2e1* expression at several CpG sites (*r* > -0.8, *P* <0.05) (Figure 7B, a, and 7C, a). At D2, unlike other REs, the average methylation over R3 showed an equally strong negative correlation with both *Mecp2e1* (*r* = -0.94, *P* <0.05) and *Mecp2e2* (*r* = -0.98, *P* <0.01) (Figure 7A, a). Therefore, we studied the two individual CpG sites located within R3 far apart from each other, which were differentially methylated (CpG1, approximately 10% and CpG2, approximately 30%) (Figure 6A, c). Interestingly, CpG1 showed a significant negative correlation with *Mecp2e1* (*r* = -0.9, *P* <0.05), while CpG2 showed a significant negative correlation with *Mecp2e2* (r = -0.9, *P* <0.01) (Figure 7B, c)*.* Further confirming the potential role of these two CpG sites within R3 in *Mecp2* isoform-specific expression, a similar correlation (CpG1: *Mecp2e1*, *r* = -0.85, *P* <0.05; CpG2: *Mecp2e2*, *r* = -0.87, *P* <0.05) was observed at D8 (Figure 7C, c). The studied intron 1 regions seemed to have preferential correlation with individual isoforms. For instance, at D2, the only CpG site within R5 showed negative, significant correlation with *Mecp2e1* (*r* > -0.8, *P* <0.05) (Figure 7B, e). Interestingly, at D8 intron 1 R4 and R6 showed positive, significant correlation with the *Mecp2e2* isoform (*r* >0.8, *P* <0.05) (Figure 7C, d-f). The observed correlations for all REs are represented in Figure 7B-C and are summarized in Figure 8.

Taken together, these results show a strong (*r* > 0.8, *P* <0.05) and dynamic (positive or negative) relationship between DNA methylation at the *Mecp2* REs and expression of *Mecp2* isoforms depending on the different stages of NSC differentiation. Therefore, these results implicate a possible dynamic role of DNA methylation at the *Mecp2* REs in regulating *Mecp2* isoform-specific expression.

## Discussion

In the brain, precisely controlled *MECP2*/MeCP2 transcript and protein expression levels are critical, as even slightly altered expression is associated with severe neurological symptoms [2,16,57-60]. However, so far little is known about how MeCP2 expression is regulated in the developing brain. MeCP2 is a major epigenetic regulator in brain, and its reduced expression in the autistic brain is associated with *MECP2* promoter hypermethylation [2]. Surprisingly, the role of DNA methylation in MeCP2 expression during brain development is unclear. Currently, most diseases that are associated with aberrant MeCP2 function or expression deficits, including autism and Rett syndrome, have no cure or effective treatment. This underscores an urgent need for investigating how MeCP2 expression is regulated in the brain. Such knowledge for addressing this gap is essential for designing possible future therapeutic strategies. DNA methylation is a reversible epigenetic modification [22], which can be targeted by existing Food and Drug Administration (FDA)-approved drugs, including decitabine, which is suggested for use in autism [61,62]. Therefore, investigating the effect of such epigenetic drugs on MeCP2 expression is important. Therapeutic approaches such as gene therapy or restoring MeCP2 expression by genetic engineering have been suggested as possible therapeutic strategies for MeCP2-associated disorders [14,15,35]. However, even mild MeCP2 overexpression can lead to severe neurological complications, highlighting the importance of understanding MeCP2 regulatory mechanisms. Since both MeCP2 isoforms have been implicated in severe neurological disorders, investigating MeCP2 regulation is equally important for individual isoforms. This present study is the first report on the potential role of DNA methylation at the *Mecp2* REs and the impact on the expression of *Mecp2* isoforms.

We observed globally altered DNA methylation upon decitabine exposure and withdrawal. Since DNA methylation is a major epigenetic mechanism that is involved in modulating gene expression and chromatin architecture [22], these observed changes in 5mC levels may possibly lead to altered chromatin structure and genome-wide changes in gene expression. Furthermore, the presented findings highlight that exposure to drugs that disturb the epigenetic marks during differentiation of brain cells may lead to aberrant DNA methylation profiles. Our observations at D8 indicate that, even after the disturbance factor is withdrawn from the system, an epigenetic memory for this disturbance may be associated throughout cellular differentiation of brain cells. Thus, our findings highlight the biological importance of maintaining proper regulation of epigenetic factors/modifications during brain development with a clear focus on DNA methylation and MeCP2.

Our results show that decitabine alters *Mecp2*/MeCP2 expression at both the transcript and protein levels. Importantly, even minor changes in *Mecp2* transcript expression led to nearly 2- to 3-fold altered protein expression, highlighting the biological significance of proper regulation of *Mecp2* expression at the transcript levels. The observed correlation between the *Mecp2*/MeCP2 (total) and *Mecp2e1*/MeCP2E1 transcript/protein expression at D2 reinforces the concept that potential changes in *Mecp2* transcript levels may reflect possible changes at the protein levels. However, the non-correlated *Mecp2*/MeCP2 (total) and *Mecp2e1*/MeCP2E1 transcript/protein expression at D8 indicates that decitabine withdrawal causes not only transcriptional but also, post-transcriptional regulation of MeCP2 expression, leading to reduced expression of MeCP2 (total)/MeCP2E1. One such post-transcriptional regulatory mechanism could be the action of micro-RNAs such as miR132, expression of which has been shown to be increased by 5-aza-2′-deoxycytidine/decitabine [63], and has the ability to repress MeCP2 expression [64].

Increased promoter methylation of autistic candidate genes such as *RORA*, *BCL2* and *MECP2* are shown to be associated with reduced expression of these genes in autistic patients [2,19,62]. Treatment with decitabine was shown to demethylate promoters and restore/induce the expression of the silenced *RORA* and *BCL2* in autistic and patients with fragile X syndrome and hence, the use of DNA demethylating agents in drug therapy for autism and fragile X syndrome has been suggested [61,62]. A similar strategy to restore/induce MeCP2 expression might be extended to treat such diseases associated with reduced MeCP2 expression, including autism and RTT. Providing insights on such therapeutic strategies, the application of epigenetic drug therapy to induce non-mutated copy of *MECP2* expression in Rett syndrome cell lines has been suggested and attempted previously [65]. Therefore, our findings on the ability of decitabine to induce MeCP2 expression in differentiating NSC provide further insights on designing possible drug therapies for autism. Even though the exposure of RTT cell lines (fibroblasts) to lower doses of decitabine for a longer period did not activate *MECP2* expression [65], our results indicate that moderate dose of decitabine can induce *Mecp2*/MeCP2 expression within a shorter period. However, inhibition of MeCP2 by withdrawal of decitabine as well as other observed changes in DNA methyl marks implies that such drug therapy should be administrated with great caution.

Our findings on the changes in DNA methylation at the *Mecp2* REs are in agreement with the previous reports on *MECP2* promoter methylation, which demonstrate that an approximate difference of 2.0 to 2.5% overall methylation over a region -233 to -531 upstream of the *MECP2* promoter is correlated with reduced *MECP2* expression in autistic male brains. The authors report that within the 15 CpG sites found in this *MECP2* promoter region, two CpG sites are specifically altered in the autistic males [2]. Furthermore, our results are in agreement with a previous report on significantly reduced MeCP2 expression in the postnatal mouse brain (under stress), which is associated with 2 to 5% increased methylation at the individual CpG sites within a 164-bp region of the *Mecp2* promoter [20]. Supporting these observations, studies have also shown minor differences, such as 2 to 5% DNA methylation causing significant changes in the expression of other genes, such as *RASSF1*, in the human brain [66], *AMOTL2* in the human heart [67], and *PGC1α* in human muscles [68]. Therefore, although the detected DNA methylation changes in this current study are not considerably high (they varied between 2 to 15%), they were statistically significant for average DNA methylation (within R1, R3 and R5) during NSC differentiation, and for several specific CpG dinucleotides subsequent to decitabine treatment (within R2, R4, R5, and R6), and are likely to be biologically important.

The *Mecp2* promoter CpG island studied by Franklin *et al*., [20] overlaps with the R1 and R2 of the *Mecp2* promoter that we studied here. The significantly methylated CpGs reported in their study coincides with the R2 CpGs, where we observed changes at individual CpG sites as well as average methylation upon decitabine treatment. However, in our study we did not see any significant change in the R1 CpG sites (both D2 and D8), where Franklin *et al*., reported DNA methylation changes. Importantly, the results we obtained for one of the promoter regions studied (R2) are in agreement with this previous report, which showed a biological and functional importance of the methylation changes in regulating MeCP2 expression in response to stress *in vivo*. Therefore, it is likely that the detected changes we observed in the *Mecp2* REs in our study also have biological importance. The hypermethylation of this R2 region in mouse brain was associated with MeCP2 downregulation [20], and hence it is possible that the hypomethylation/demethylation of the same R2 region causes *Mecp2/*MeCP2 upregulation.

Our results on the ability of 2.5 μM decitabine to upregulate *Mecp2e1* (but not *Mecp2e2*) suggest that the two isoforms may have different sensitivities to drugs/chemicals. This observation is in agreement with the previous report on the higher sensitivity of *Mecp2e1* than *Mecp2e2* to Bisphenol A [69]. These observations further suggest that the differential sensitivity to drugs might be used to specifically induce only one *Mecp2* isoform. This is also important because overexpression of *Mecp2e2,* but not *Mecp2e1* causes neuronal cell death [10]. Hence, our study provides a functional relevance of DNA demethylation at the *Mecp2* REs by decitabine causing upregulation of *Mecp2e1*, but not *Mecp2e2*.

The observed negative correlation between the expression of both *Mecp2* isoforms and *Mecp2* promoter elements are novel and are in accordance with previous correlation studies on the human *MECP2* expression and promoter DNA methylation [2,19]. Furthermore, our study is novel in demonstrating a dynamic (positive/negative) correlation between the intronic DNA methylation and expression of *Mecp2* isoforms in differentiating brain cells. It is possible that the promoter regions analyzed in our study (which also overlap with the core *Mecp2* promoter [13]) might be shared by both *Mecp2* isoforms, whereas depending on the stage of neural differentiation, intron 1 regions may add another layer of regulation for *Mecp2* isoform-specific expression. Supporting our findings, the role of intronic DNA methylation in regulating gene expression of other genes has been previously reported [70,71]. Several other reports also show evidence that gene expression negatively correlates with promoter methylation and positively correlates with gene-body methylation [67,72].

Intronic DNA methylation is reported to be involved in regulating alternative splicing [27,28]. Although, it is known that *Mecp2* isoforms are generated by alternative splicing [4,5], the underlying molecular mechanisms are still unclear. We observed that the expression ratio of *Mecp2e1/Mecp2e2* changed during NSC differentiation. The observed correlation between the splice ratio and intron 1 R4 DNA methylation in differentiating NSC at D2 would provide insights on the potential importance of this region in *Mecp2* alternative splicing.

The intron 1 regions analyzed in this study were designated as part of a silencer element, which has been previously proposed to regulate *MECP2* alternative splicing and tissue-specific expression [12]. Our findings are in agreement with possible involvement of these regions in *Mecp2* isoform-specific expression. Although the link between DNA methylation and *Mecp2* expression is supported by our results in the NSC system, the contribution of other epigenetic modifications such as histone acetylation and histone methylation should not be excluded [73,74].

## Conclusion

The summary of the findings presented in our study is illustrated in Figure 8. First, expression of *Mecp2* isoforms was significantly and reciprocally changed at different stages of NSC differentiation, in association with minor but significant changes in DNA methylation at selected *Mecp2* REs, suggesting possible involvement of these regions in *Mecp2* regulation. Second, treatment of differentiating NSC with decitabine for 48 h led to demethylation of specific *Mecp2* REs (promoter R2 and all intron 1 regions) and subsequent upregulation of *Mecp2e1/*MeCP2E1 (but not *Mecp2e2*), implying the differential sensitivity of the two *Mecp2* isoforms to decitabine. Such differential sensitivity of *Mecp2* isoforms to decitabine might be useful in future drug therapies to specifically activate one isoform but not the other. Furthermore, the ability of decitabine to induce *Mecp2e1*/MeCP2E1 at both transcript and protein levels provide insights for future therapeutic strategies for MeCP2 deficiency-related neurodevelopmental disorders such as autism and Rett syndrome. Finally, the significant and dynamic (positive or negative) correlation between the expression of *Mecp2* isoforms and DNA methylation implies the potential contribution of these REs in regulating *Mecp2* isoforms at different stages of neural differentiation. Collectively, our study contributes to the understanding of expression and regulation of *Mecp2* isoforms during neural development and provides important insights for future therapeutic applications of decitabine for MeCP2-related neurological disorders.

## Abbreviations

5hmC: 5-hydroxymethylcytosine; 5mC: 5-methylcytosine; ANOVA: Analysis of variance; bFGF: Basic Fibroblast growth factor; bp: Base pairs; CNPase: 2',3'-Cyclic-nucleotide 3'-phosphodiesterase; D: Day; DAPI: 4',6-diamidino-2-phenylindole; DMEM: Dulbecco's modified Eagle’s medium; DNMT: DNA methyltransferases; E: Embryonic day; GAPDH: Glyceraldehyde-3-phosphate dehydrogenase; GFAP: Glial fibrillary acidic protein; IF: Immunofluorescence; MBP: Myelin basic protein; MECP2: Methyl CpG binding protein 2, human gene; Mecp2: Methyl CpG binding protein 2, mouse gene; MeCP2: Methyl CpG binding protein; NEUN: NEUronal Nuclei; NSC: Neural stem cells; OLIG2: Oligodendrocyte lineage transcription factor 2; qRT-PCR: Quantitative reverse transcription polymerase chain reaction; r: Pearson’s correlation coefficient; RE: Regulatory element; RTT: Rett syndrome; rhEGF: Recombinant human epidermal growth factor; SEM: Standard error of the mean; TUB III: TUBULIN III; WB: Western blot.

## Competing interests

The authors have declared that no competing interests exist.

## Author contributions

VRBL, RMZ and MR conceived and designed experiments. MR performed neural stem cell isolation, culture and differentiation. RMZ maintained neural stem cell cultures. VRBL performed qRT-PCR, dot blot, WB, IF, inverted microscopy imaging. RMZ performed IF and confocal imaging. VRBL and MR analyzed data. MR contributed reagents/materials/analysis tools. VRBL and MR wrote the paper. All authors read and approved the final manuscript.

## Supplementary Material

Additional file 1: Figure S1 Comparison of CpG sites in human Methyl CpG binding protein 2 gene (*MECP2*) and mouse Methyl CpG binding protein 2 gene (*Mecp2*) promoter and intron 1. CpG sites analyzed in the mouse *Mecp2* (black) are underlined. Conserved CpGs between mouse and human sequences are also underlined in human *MECP2* (red) sequence. Click here for file

Additional file 2: Figure S2 Detection of KI67 in day 2 (D2) control cells. KI67 was detected in (98.8% ± 0.8) of the D2 cell population, indicating that they were actively proliferating. Scale bars represent 20 μm. Click here for file

Additional file 3: Figure S3 Relationship between the ratio of mouse Methyl CpG binding protein 2 gene *Mecp2* splice variants and DNA methylation at selected *Mecp2* regulatory elements. Pearson’s correlation analysis between DNA methylation at the *Mecp2* regions R1, R4 and R5 and *Mecp2e1/Mecp2e2* ratio at different stages of neural stem cell (NSC) differentiation. Significant differences: ^*^P <0.05. The regions are, promoter regions R1: CpG island contains 13 CpG sites, intron 1 regions R4: 1 CpG site, and R5: 1 CpG site; n = 3 ± standard error of the mean. Click here for file

Additional file 4: Figure S4 Determination of the male/female contribution at different stages of neural stem cell (NSC) differentiation. (A) PCR amplification of *Sry* (402 bp) and *Il3* (544 bp) in adult male cortex (positive control) and the absence of the signal in negative control PCR (no template). (B) The detection of *Sry* and *Il3* in the positive and negative controls and during NSC differentiation (day 0 (D0), D2, D8). The graph represents ratio of *Sry*/*Il3*; n = 3 ± standard error of the mean (SEM). (C) Expression of *Xist* transcripts relative to *Gapdh* at different stages of NSC differentiation; n = 3 ± SEM. Significance was determined at ^*^P <0.05. (D) Ratio of *Sry*/*Il3* in D2 control and D2 decitabine-treated cells. (E) Ratio of *Sry*/*Il3* in control and decitabine-treated cells at D8; n = 3 ± SEM. Expression of *Xist* transcripts relative to *Gapdh* at D2 after decitabine treatment (F), and at D8 after decitabine withdrawal (G); n = 3 ± SEM. Significance was determined at *P <0.05. Click here for file
