# Supplementary figures and images for: Decitabine alters the expression of Mecp2 isoforms via dynamic DNA methylation at the Mecp2 regulatory elements in neural stem cells
Source: Mol Autism. 2013 Nov 15;4:46. doi: 10.1186/2040-2392-4-46 (PMC3900258; doi:10.1186/2040-2392-4-46)

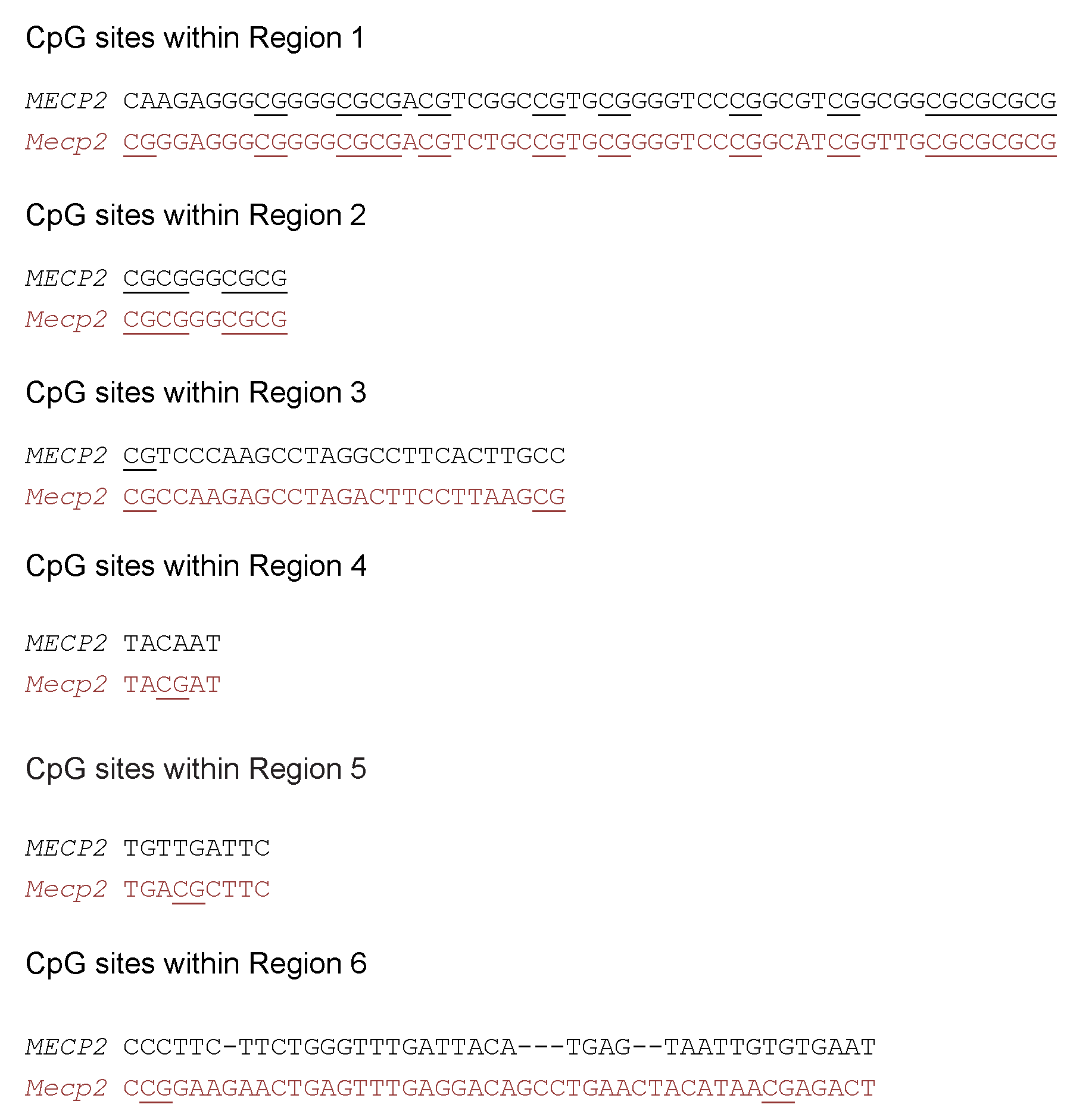

Supplement: Additional file 1: Figure S1 — Comparison of CpG sites in human Methyl CpG binding protein 2 gene (MECP2) and mouse Methyl CpG binding protein 2 gene (Mecp2) promoter and intron 1. CpG sites analyzed in the mouse Mecp2 (black) are underlined. Conserved CpGs between mouse and human sequences are also underlined in human MECP2 (red) sequence. [file 2040-2392-4-46-S1.tiff]

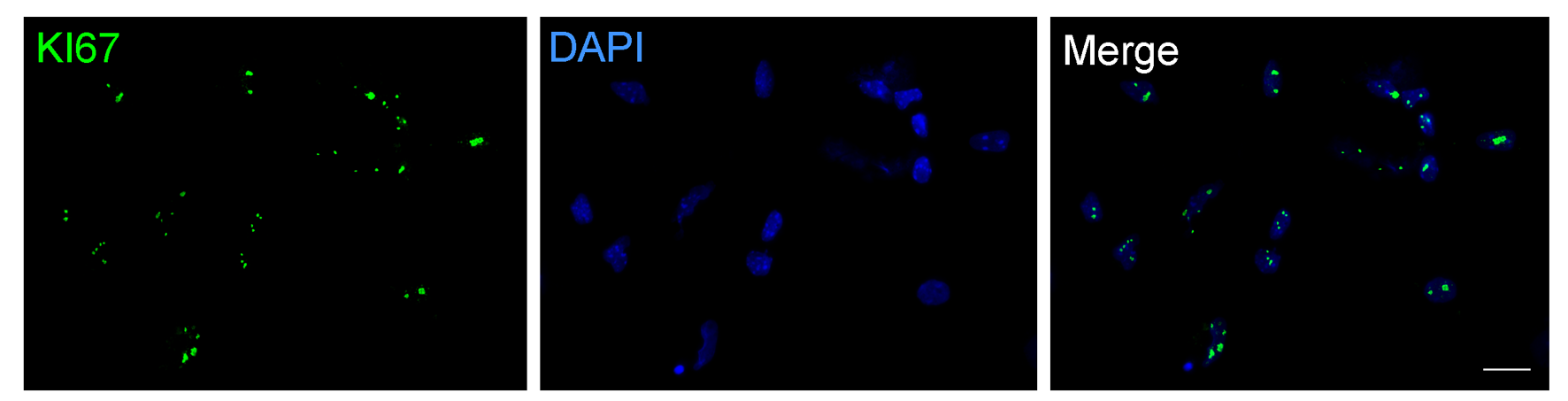

Supplement: Additional file 2: Figure S2 — Detection of KI67 in day 2 (D2) control cells. KI67 was detected in (98.8% ± 0.8) of the D2 cell population, indicating that they were actively proliferating. Scale bars represent 20 μm. [file 2040-2392-4-46-S2.tiff]

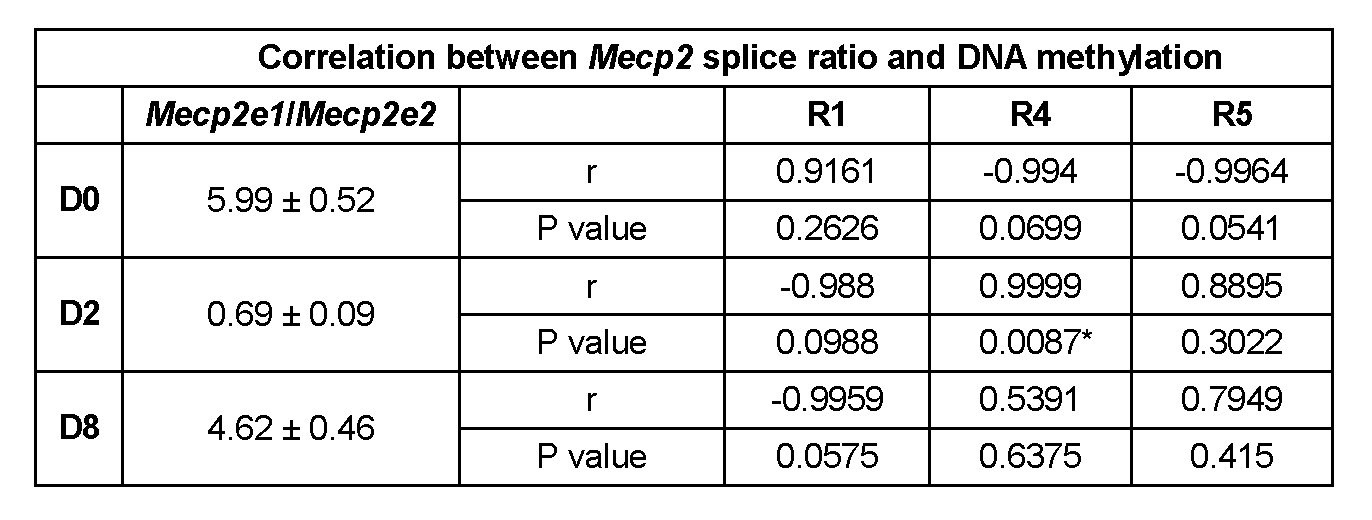

Supplement: Additional file 3: Figure S3 — Relationship between the ratio of mouse Methyl CpG binding protein 2 gene Mecp2 splice variants and DNA methylation at selected Mecp2 regulatory elements. Pearson’s correlation analysis between DNA methylation at the Mecp2 regions R1, R4 and R5 and Mecp2e1/Mecp2e2 ratio at different stages of neural stem cell (NSC) differentiation. Significant differences: *P <0.05. The regions are, promoter regions R1: CpG island contains 13 CpG sites, intron 1 regions R4: 1 CpG site, and R5: 1 CpG site; n = 3 ± standard error of the mean. [file 2040-2392-4-46-S3.tiff]

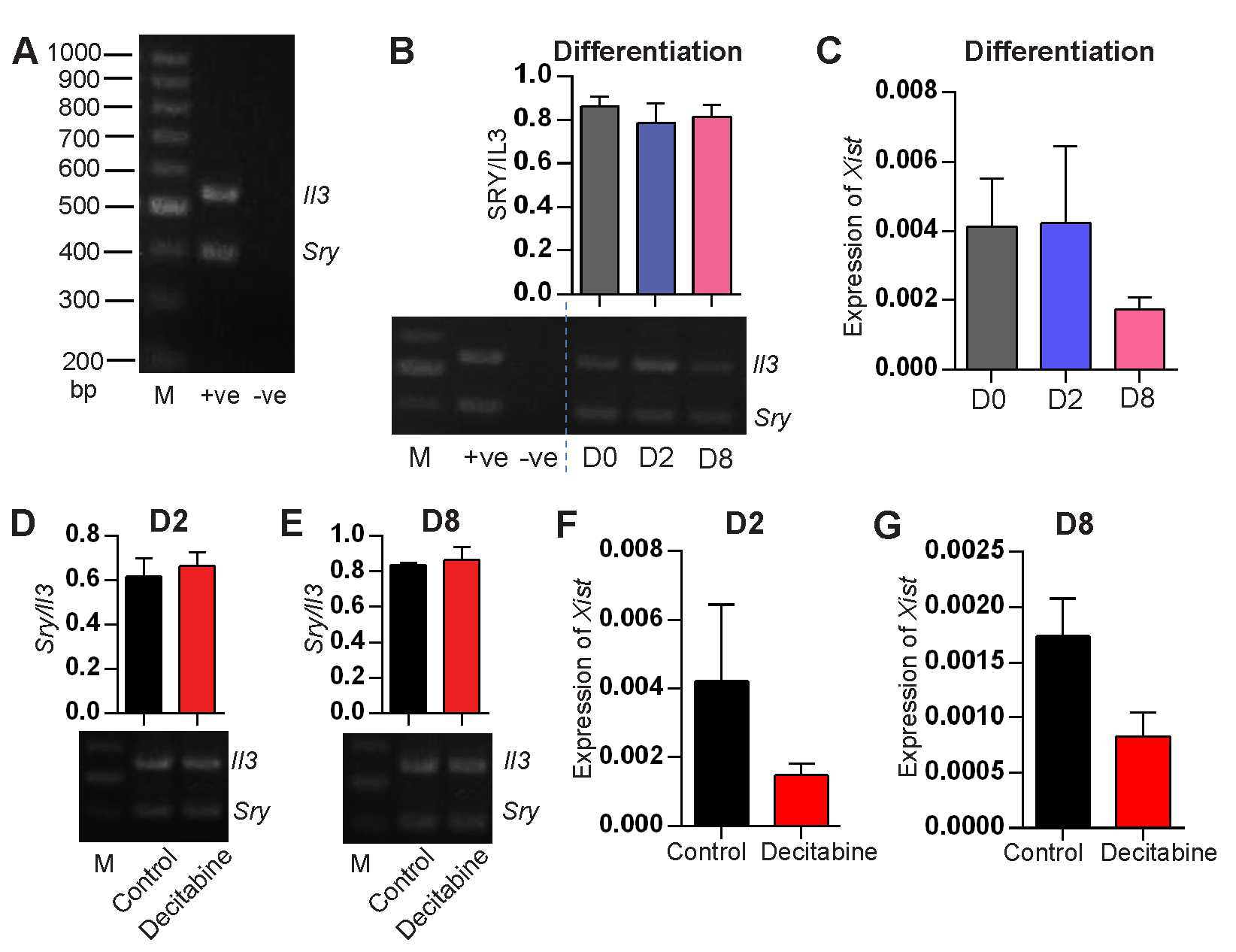

Supplement: Additional file 4: Figure S4 — Determination of the male/female contribution at different stages of neural stem cell (NSC) differentiation. (A) PCR amplification of Sry (402 bp) and Il3 (544 bp) in adult male cortex (positive control) and the absence of the signal in negative control PCR (no template). (B) The detection of Sry and Il3 in the positive and negative controls and during NSC differentiation (day 0 (D0), D2, D8). The graph represents ratio of Sry/Il3; n = 3 ± standard error of the mean (SEM). (C) Expression of Xist transcripts relative to Gapdh at different stages of NSC differentiation; n = 3 ± SEM. Significance was determined at *P <0.05. (D) Ratio of Sry/Il3 in D2 control and D2 decitabine-treated cells. (E) Ratio of Sry/Il3 in control and decitabine-treated cells at D8; n = 3 ± SEM. Expression of Xist transcripts relative to Gapdh at D2 after decitabine treatment (F), and at D8 after decitabine withdrawal (G); n = 3 ± SEM. Significance was determined at *P <0.05. [file 2040-2392-4-46-S4.tiff]
